# Rotaviruses: From Pathogenesis to Disease Control—A Critical Review

**DOI:** 10.3390/v14050875

**Published:** 2022-04-22

**Authors:** Cornelius A. Omatola, Ademola O. Olaniran

**Affiliations:** Discipline of Microbiology, School of Life Sciences, College of Agriculture, Engineering and Science, Westville Campus, University of KwaZulu-Natal, Private Bag X54001, Durban 4000, South Africa; 220113323@stu.ukzn.ac.za

**Keywords:** rotavirus, pathogenesis, diarrhea, epidemiology, vaccination

## Abstract

Since their first recognition in human cases about four decades ago, rotaviruses have remained the leading cause of acute severe dehydrating diarrhea among infants and young children worldwide. The WHO prequalification of oral rotavirus vaccines (ORV) a decade ago and its introduction in many countries have yielded a significant decline in the global burden of the disease, although not without challenges to achieving global effectiveness. Poised by the unending malady of rotavirus diarrhea and the attributable death cases in developing countries, we provide detailed insights into rotavirus biology, exposure pathways, cellular receptors and pathogenesis, host immune response, epidemiology, and vaccination. Additionally, recent developments on the various host, viral and environmental associated factors impacting ORV performance in low-and middle-income countries (LMIC) are reviewed and their significance assessed. In addition, we review the advances in nonvaccine strategies (probiotics, candidate anti-rotaviral drugs, breastfeeding) to disease prevention and management.

## 1. Introduction

Globally, approximately 258 million cases of infectious diarrhea in under five years children are attributable to rotavirus (RV) infection [1]. Between 2013 and 2017, an estimated 122,000–215,000 diarrheic child deaths were caused by RV annually [1,2,3]. Among all causes of death in under five-year children, RV has been rated the third leading pathogen associated with childhood mortality [3]. Children in low- and medium-income countries (LMIC) compared to high-income countries (HIC) particularly bear the brunt of the diarrheal deaths [1]. A global health-related statistic in 2016 shows that approximately 100 per 100,000 children die before their fifth birthday in all 10 developing countries (India, Pakistan, Kenya, Democratic Republic of Congo, Niger, Angola, Ethiopia, Afghanistan, Nigeria, and Chad) bearing the highest RV diarrheic burden [2]. Four oral, live vaccines have since been prequalified by WHO and licensed internationally [4]. Despite the successful implementation of the vaccines in over 106 countries, RV is still responsible for the highest number of annual childhood deaths attributable to diarrhea globally [5].

Several factors associated with the human host (e.g., malnutrition, histo-blood group antigens, ORV co-administration with polio vaccine and maternal factors), agents (e.g., genetic diversity, the force of infection and co-infection), and environment (enteropathy or dysbiosis of gut microbiome) have been suggested as possible etiologies driving the differences in vaccine-elicited protective immunity between the two socioeconomic settings [6,7]. With the emerging insights into structure–function relationships of RV proteins versus interactions with the host, this article provides information on past and present knowledge on the viral biology, pathogenic mechanisms, innate and adaptive immune response of the host, diagnosis, epidemiology and genetic diversity, and disease control through vaccination. Additionally, we critically appraise the progress in understanding the epidemiological triad of vaccine underperformance in developing countries.

## 2. Etymology and Biology of Rotavirus

Rotavirus (RV) was first discovered in the 1950s in rectal swabs of monkeys and later in the 1960s in intestinal biopsy of mice by electron microscopy [8]. In 1973, Ruth Bishop and colleagues first described the virus in children presenting with gastroenteritis [9]. A year later, rotavirus was detected in large quantities in fecal samples from hospitalized children with acute nonbacterial gastroenteritis by direct thin-layer electron microscopy and by immune electron microscopy [10]. The viral particle from children was initially referred to by several names, including reovirus-like, orbivirus-like, duovirus, infantile gastroenteritis virus, or a ‘new’ virus. In 1974, Thomas Henry Flewett suggested the name *rotavirus* because of its characteristic wheel-like appearance when observed under the electron microscope [11]. Four years later, the name *rotavirus* was officially accepted by the International Committee on Taxonomy of Viruses. The detection of rotaviruses in several other species of animals led to them being recognized as pathogens affecting humans and animals worldwide [11]. Within just 5 years of discovery, rotavirus became recognized as one major etiology of diarrhea in infants and young children globally, accounting for approximately one-third of cases of severe diarrhea requiring hospitalization [12]. Up to the present, rotavirus has remained the leading cause of acute infectious gastroenteritis in infants and young children with a high rate of hospitalization and death globally [13,14].

**Figure 1:**
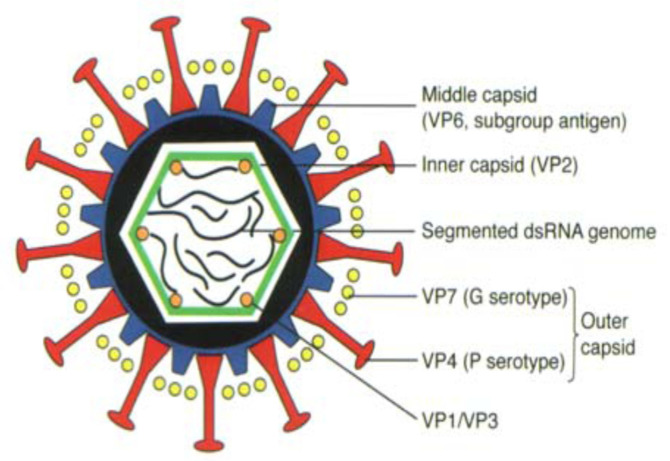
Schematic representation of the rotavirus virion [24].

Rotavirus is a member of the Reoviridae family and three types of particles (double-shelled, single-shelled, and core) arranged in concentric rings, formed a triple-layered particle (TLP) around the genome, which becomes the infectious form of the virus [15]. The double-shelled, single-shelled, and core particles are 76.5 nm, 70.5 nm, and 50 nm in diameters, respectively (Figure 1). The genome of rotavirus consists of 11 segments of double helix molecules of RNA, which code for six structural viral proteins (VP1, VP2, VP3, VP4, VP6, and VP7) and six non-structural proteins (NSP1, NSP2, NSP3, NSP4, NSP5, and NSP6) [16,17]. The major antigenic properties of the rotaviruses group, subgroup, and serotype are determined by the viral capsid proteins (VPs) [18]. The NSPs are produced during infection to facilitate viral replication and pathogenesis [19]. The specific roles of the VPs and NSPs are indicated in Table 1.

The VP7 and VP4 of rotavirus are employed in binary classification systems to delineate rotavirus into G (glycoprotein) and P (protease-sensitive) genotypes, respectively [20]. To date, 36 G-types and 51 P-types have been described in different surveillance studies in both humans and animals across the globe [21] (Table 1). More recently, the binary strain typing system was replaced by a whole genome or 11-gene typing system to ascribe genotypes to each gene: Gx-P[x]-Ix-Rx-Cx-Mx-Ax-Nx-Tx-Ex-Hx, which codes for the VP7-VP4-VP6-VP1-VP2-VP3-NSP1-NSP2-NSP3-NSP4-NSP5/6, respectively [22]. Three genotype constellations have been described for human rotavirus genomes, namely; Wa-like (genogroup 1; G1/3/4/9/12-P[8]-I1-R1-C1-M1-A1-N1-T1-E1-H1), DS-1-like (genogroup 2; G2-P[4]-I2-R2-C2-M2-A2-N2-T2-E2-H2), and AU-1-like (genogroup3; G3-P[9]-I3-R3-C3-M3-A3-N3-T3-E3-H3) [23]. Unlike the more common Wa-like and DS-like, the AU-1-like are reported infrequently in humans [23].

## 3. Exposure Pathways in Developing Countries

**Figure 2:**
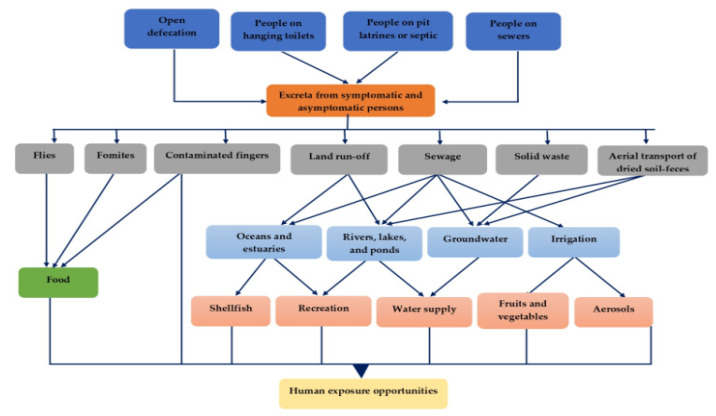
Exposure pathways of rotavirus (conceptualized from refs. [28,68,69]).

The transmission of RV follows the fecal–oral route [27] (Figure 2). The fecal–human spread is mainly facilitated by environmental reservoirs such as fluids, food, fingers, and fomites through interactions of humans or animals with their environments [28]. In addition, flies as a natural process can also spread RV shed in feces. Spreads of the virus are quite easy among children and from infected children; transmission to close contacts is possible. In affected persons, acute illness is usually characterized by the early stage of the disease, which subsequently results in milder illness with no visible symptoms in some individuals. In adults, asymptomatic infections can lead to viral transmission to close contacts [29]. The frequent exposure of susceptible children in the day-care centers and family day-care homes usually facilitates RV transmission [30]. The findings of rotavirus on diaper disposal containers, toys, faucets, diaper changing areas, handwashing areas, and even in food preparation areas are suggestive of its high potential of spread throughout most homes or day-care centers [31,32]. Children are often seen putting toys into their mouths while playing or scratching their gums with them when they are near to start teething. Such objects when contaminated can efficiently transmit RV in the process [33]. A nosocomial RV outbreak associated with sharing of toys among children in a pediatric oncology unit hospital has been reported [34]. Asymptomatic children tend to have lower viral shedding with a likelihood of intermittent shedding than children already presenting with diarrhea [32]. Both asymptomatic and symptomatic health care workers have been linked to the spread of the virus in some outbreaks. Rotavirus is a ubiquitous and vastly stable organism that may persist in the environment for weeks or months without losing infectivity if not disinfected [35]. The doggedness of RV infectivity on porous (paper and cotton cloth) and nonporous surfaces (aluminum, latex) have been documented [36]. Viral transmission and ubiquity are potentiated by the low infectious dose (<100 viral particles), high concentration of virus in the stool (1012 particles per gram), and protracted shedding of virus [13].

Fecal contamination of food leading to foodborne illnesses has been tagged as an efficient system for transmission of RV [28]. Food contamination usually occurs when polluted water or inefficiently treated sewage sludge and effluents are used for irrigation of crops, or food handlers fail to ensure proper hand hygiene [28,37]. Quiroz-Santiago et al. [38] detected RVA in oysters and also reported its occurrence in 21.2% (7/33) of vegetable samples comprising celery, coriander, spinach, romaine lettuce, papaloquelite, and parsley, which were brought to a Mexican market. Similarly, RVA was detected in partially treated water (11.8%), irrigation water (14%), and the corresponding raw vegetable samples (1.7%) in Southern Africa [39]. Genotyping studies further revealed clinically relevant VP 7 (G) strains (G1, G2, G8, and G9) and VP4 (P) types (P[4], P[6], P[8], and P[9]). There have been several reports of foodborne rotavirus gastroenteritis outbreaks in association with contaminated food. For instance, a national health report in Japan attributed the RV outbreak that occurred among the adult population to eating at a restaurant [40]. Food vehicles that have been implicated in RV outbreaks included crustaceans [41,42], tuna and chicken sandwiches [43], cabbage [44], salads [45], and a potato stew [46].

The vehicular role of human fingers in the spread of rotaviral infections has been demonstrated in the literature. Infectious RV particles placed on human fingers were shown to persist for more than 60 min without inactivation and could contaminate environmental surfaces following contact [47,48]. This observation shows that human fingers when contaminated pose both direct and indirect diarrheal disease risks. The direct risks usually involve hand-to-mouth contacts. Previous studies have shown that children and adults touch their mouths approximately 3–28 and 8 times, respectively, every hour [49]. The higher the frequency of contacts, the greater the risk of exposure [28]. One significant predictor of rotavirus dissemination and positivity in vulnerable contacts is the failure to wash a child’s hands after every visit to the toilet or before a meal [50]. The indirect risks on the other hand involve the transfer of infectious viruses from contaminated hands to fomites, drinking water, and food during contacts. Notably, this exposure pattern is exemplified by the role infected food handlers play in RV transmission. For instance, an outbreak of Group A rotavirus (RVA) gastroenteritis that occurred among University students in Washington, District of Columbia, was connected with eating deli sandwiches that were contaminated by an infected food handler [43]. Additionally, food handlers were associated with two confirmed isolated rotavirus gastroenteritis outbreaks involving 28 school children in Colorado in 2009 and 30 children and adults at a banquet facility in New York in 2005, which were reported to CDC by Foodborne Disease Outbreak Surveillance System [51].

Flies are naturally attracted to both feces and food, making them important reservoirs for RV and other enteric pathogen transmissions [28]. Rotaviruses in feces are naturally picked up either through direct contact with the fly exoskeleton or consumption of the feces. Contamination of surfaces such as food, fomites, or skin may occur mechanically following the transfer of RV from the exoskeleton or through regurgitation and fecal deposits [52]. The high number of flies often seen in areas of human activities such as restaurants, food markets, fish markets, slaughterhouses, and hospitals have been correlated with significant risks of viral transmission and infection when eventually transferred to the mouth and ingested [53]. A previous investigation of foodborne gastroenteritis outbreaks in India detected rotaviruses in 6.7% of fly samples trapped from household kitchens, suggesting the potential for mechanical transmission [54].

Rotaviruses of animal origin can infect humans either by direct transmission of the virus or through the contribution of one or more of the genomic RNA segments to form reassortants with mosaic gene constellations of human and animal RVA origin genes [55]. Currently, some of the unusual RV genotypes that have been identified in humans were shown to be a result of animal–human transmission [56]. Rotavirus remains an important cause of diarrhea in wild animals (llamas, giraffes), farm animals (pigs, sheep, and cows), rodents, birds, and domestic pets (cats and dogs) worldwide, and the availability of animal hosts represents a potential reservoir for genetic exchange with human rotavirus strains [57].

Rotavirus transmission through sewage or sewage polluted river water is on the increase in developing countries due to high population growth and poor sanitary conditions [58]. RV occurrence in finally treated drinking water is also a challenge [39]. The ability to persist for days in water environments and be infectious facilitates waterborne spread, which has resulted in several gastroenteritis outbreaks with a wide range of symptoms [27]. Drinking water has been regarded as the most efficient exposure pathway for RV infection and as a shared resource, contamination by one member of the household can amplify the risks of transmission to all susceptible contacts in a home [28]. Each day, the average child and adult consume approximately 0.2–0.5 and 0.8–1.2 L of water, respectively [49]. Even when the source water is considered safe, the same might not apply to stored water because of the ubiquitous nature of RV. Contamination of water storage containers, hands, drinking cups, and other utensils is common in most homes, and their contact with stored water has been implicated in the degradation of water quality in the home [59].

The airborne spread of rotavirus infection has also been hypothesized because of the short incubation period (1–3 days), rapid seasonal transmission through populations, and the massive nature of outbreaks [60]. However, this has not yet been established in humans. Aerosol transmission of RV had been suggested in connection with simultaneous outbreaks that occurred in isolated communities on Native American reservations and in Aboriginal infants in Central Australia [61,62]. The detection of RV in respiratory secretions and cases of pneumonia among a small number of patients has been documented [63,64]. Additionally, respiratory symptoms and otitis media have been described in approximately 50% of patients with rotavirus infection [65,66]. While attempting to confirm the potentiality of aero-transmission, Wilde et al. [31] employed the highly sensitive RT-PCR to confirm the presence of RV RNA in air samples from the rooms of hospitalized children with acute rotavirus infections. More recently, Ginn et al. [67] detected genes specific to rotavirus in aerosol samples from near open wastewater with poor sanitation facilities. While these are informative, further investigations into the viability of the aerosolized virus are needed to further confirm airborne transmission.

## 4. Pathogenesis of Rotavirus Infection

### 4.1. Viral Entry and Site of Primary Replication

The triple-layered capsid structure of RV confers relative stability on the virion and facilitates fecal–oral transmission as well as efficient delivery into the small intestine without inactivation [70,71]. Rotaviruses target and infect mature, non-dividing absorptive villous epithelium of the upper two-thirds of the small intestine [72]. The enteroendocrine cells have also been shown to be susceptible to infection [19]. The initial viral–host interaction is facilitated by the binding of outer capsid protein VP4 (through its VP8* domain) and host cell receptors, which include the sialoglycans (such as gangliosides GM1 and GD1a) and histo-blood group antigens (HBGAs) [19,73,74]. The interaction via polymorphic HBGA in red blood cells, mucosal secretions, and epithelia is biased by a particular rotavirus P genotype [75,76]. The HBGA are complex glycans that are catalyzed by glycosyltransferases through series of monosaccharides addition to an initial precursor. The enzyme expression is controlled by the *AB0*, *FUT2 (secretor),* and *FUT3 (lewis)* genes, and both in vivo and in vitro studies have demonstrated their presence as a marker of host susceptibility to several infectious diseases including group A RV [77]. The genetic differentials in HBGA expression have been likened to variations in rotavirus epidemiology among human populations [19] and infection with different RV genotypes [78,79]. For example, genotype P[8] and P[4] preferentially bind to the Lewis b and H type-1 (H1) antigens [80], genotypes P[9], P[14], and P[25] bind to type A antigens [81], while P[11] selectively binds to the type-2 precursor glycan [82]. Findings from a meta-analysis indicated a strong association between HBGA expression and susceptibility to natural infection by P[8] rotaviruses [83]. In a recent study of a rotavirus outbreak in a middle school in China, Guo et al. [84] identified a single G9P[8] rotavirus strain that only infected HBGA secretor individuals. Furthermore, a recent study by Cantelli et al. [85] showed HBGAs secretor individuals were more susceptible to rotavirus vaccine strains compared to non-secretors who lack expression of certain HBGA molecules essential for infectivity by several RV strains. This further confirms the roles HBGA plays in viral replication and also suggests its potential effect on the effectiveness of the oral rotavirus vaccines.

In the post-attachment stage, the trypsin-like proteases of the gastrointestinal tract proteolytically cleave VP4 spike into VP8 and VP5, a highly ordered conformational change in the capsid proteins and an important event that accelerates viral penetration, thus promoting infectivity [86]. Recently, the result of an electron cryomicroscopy showed VP4 activation via trypsin cleavage to VP8* and VP5* triggers its functional refolding on the virion surface from an upright to a reversed conformation. Such reversal exposes the previously buried foot domain for interaction with the host cell membrane [87].

### 4.2. Local Intestinal Infection and Disease Mechanisms

RV infection is largely localized to the intestinal mucosa, although evidence of viral replication has been shown in some distant areas of the body such as lamina propria and regional lymphatics, especially among the immunocompromised individuals. Viral replication at these extraintestinal sites and systemic spread is usually rare in immunologically competent persons [88]. Rotaviral diarrhea is caused by multiple activities of the virus. One mechanism is that the extensive replication of the virus coupled with massive cellular necrosis of the gut epithelium causes villous atrophy, loss of microvilli, severe mononuclear cell infiltration, endoplasmic reticulum and mitochondrial engorgement in enterocytes, and loss of intestinal brush border enzymes such as maltase, sucrase, and lactase [9,89]. The result of this is nutrients (D-xylose and lactose in the vicinity of acute infection), electrolytes, and fluid malabsorption leading to increased osmotic pressure in the gut lumen and subsequently onset of diarrhea [70,89,90]. Reactive crypt-cell hyperplasia following the process may accelerate rates of fluid secretion, thereby increasing the severity of diarrhea.

A second mechanism underlying RV diarrhea is that viral enterotoxin NSP4 produced by RV-infected cells binds to intestinal epithelial cells [91] and signals through phospholipase C, thereby activating signaling pathways that can induce age- and calcium ion-dependent chloride secretion into the intestinal lumen [92]. High chloride ion concentration provides an osmotic gradient that favors the movement of water into the intestinal lumen that ultimately results in secretory diarrhea [19]. The NSP4 protein inactivates the Sodium-Glucose-Lactose-Transporter proteins system (SGLT1) that mediates reabsorption of water, sugar, and body electrolytes, thereby reducing the activity of brush-border membrane disaccharidases and perhaps activation of the calcium ion-dependent secretory reflexes of the enteric nervous system as well as the loss of water from the body [70]. Under normalcy, healthy enterocytes secrete lactase into the small intestine that helps in lactose metabolism, but children with rotavirus infection are unable to tolerate milk due to lactase deficiency that can last for several weeks [93]. Such a child may experience recurrence of diarrhea after milk reintroduction into the child’s diet as a result of bacterial fermentation of the lactose in the gut [94].

A third mechanism is based on the stimulation of the enteric nervous system by the viral enterotoxin. The NSP4-mediated increase in intracellular calcium concentration induces the secretion of 5-hydroxytryptamine (5-HT) also called serotonin from enteroendocrine cells in humans. This chemical triggers the activation of enteric nerves that innervate the small intestine, thereby increasing intestinal motility, which is associated with diarrheal onset [95]. Evidence studies have shown that drugs that block such stimulation were associated with the alleviation of diarrhea [19,96].

The mechanisms that trigger vomiting usually seen in an early illness may be the result of early cytokine release acting centrally, or delayed gastric emptying [97]. Whether the latter is a result of an increase in gastrointestinal hormones (e.g., secretin, gastrin, and cholecystokinin) or vagal nerves activation associated with rotavirus infection remains an area for future study to look at. As reported by Marie et al. [98], the viral toxin can stimulate a sensory cell called enterochromaffin cells that lined the gut walls to release serotonin, a signaling substance that in turn activates the vagal afferent nerves linked to the brain’s vomition center. The vagus nerve has certain neurons that extend from the gut to the brain and vice versa. It constitutes an important signaling pathway for emetic stimuli and the generation of vomiting [96].

### 4.3. Systemic Infection

RV infection has been linked to systemic diseases such as seizures in the CNS, acute cerebellitis, and autoimmune pathology with clinical and pathophysiological implications beyond the gut [99,100]. One suggested mechanism of viral spread from the gut to CNS is that viral attachment to specific surface receptors such as histo-blood antigens, sialic acids, and integrins may be followed by the crossing of the blood–brain barrier [101]. The CNS has been identified as one of the main targets of extraintestinal infection, a reason Rivero-Calle et al. [101] attributed to rotavirus tropism toward the neuronal cells. Antigenemia and viremia are commonly found in children infected with RV even when diarrhea is not detected [102]. Patient with such condition has manifested with increased severity in terms of fever, vomiting or convulsion [103], although the underlying mechanism is yet to be unraveled. The relative importance of viremia and extraintestinal infection is more pronounced in immunocompromised patients [99]. A condition of antigenemia usually occurs on the first day of illness, intensifies between the first and third days of the appearance of symptoms, and drops afterward. Persistent antigenemia lasting for up to 11 weeks has been documented [103].

### 4.4. Host and Viral Factors Influencing Pathogenesis

Persons infected with rotavirus may be asymptomatic or symptomatic, the outcome of which is determined by a variety of viral and host factors. Age is the most significant host factor that influences the clinical outcome of RV infection. Thus, neonates infected with rotavirus do not always manifest symptoms of the disease as they are protected by maternal antibodies acquired through the placenta [104]. The decline in the level of maternal antibodies is usually coincident with the age of the highest susceptibility of infants to severe cases of rotavirus disease. This susceptibility to infection continues up to age five years before the baby begins to develop a strong immunity to the virus infection. Adults are also infected with rotavirus although severe symptomatic disease is not common. Adults’ infection may occur due to infections with an unusual virus strain or exposure to very high doses of virus [70]. Children experiences repeated exposures from birth to old age, though natural and/or vaccine-induced immunity usually makes further infections mild or asymptomatic following natural infection or vaccination [105]. Malnutrition is another factor that potentiates the severity of rotavirus diarrhea by delaying the restoration of the damaged intestinal epithelial barrier and also modifying the intestinal inflammatory responses [106]. In animal models, malnutrition superimposed with RV infection has also been shown to be associated with an enhanced viral shedding and intestinal microbiota translocation to systemic organs due to the compromised intestinal epithelial barrier [106].

The determinant of virus virulence is a function of the proteins coded by a subset of the 11 viral genes. Several of the gene segments (3, 4, 5, 9, and 10) encode proteins that regulate the multigenicity of the virus virulence. For instance, gene 3 encodes the capping enzyme that facilitates viral RNA replication in infected cells, gene 4, as well as 9, synthesize the outer capsid proteins necessary to initiate infection, gene 10 encodes a nonstructural protein (NSP4), which regulates the internal calcium homeostasis, facilitates virus replication and also functions as an enterotoxin [70]. The NSP1 protein product of gene 5 is associated with host interferon responses inhibition by mediating the breakdown of interferon regulatory factors IRF3, IRF5, and IRF7 [107].

### 4.5. Clinical Features

The clinical course of rotavirus infection varies from mild, watery diarrhea to severe, dehydrating diarrhea with vomiting and fever, sometimes leading to death [108]. The incubation period is usually between 18 to 36 h, and this may be followed normally by an acute onset of fever and vomiting [109]. Diarrhea is then seen, and this may last for five to seven days. Daily, fewer than 10 non-bloody but mucusy bowel movements are seen [110]. Bloody diarrhea has also been reported in a few cases [50]. Patients may also experience loss of appetite and dehydration. Decreased urination, dry mouth, and throat feeling dizzy when standing up, crying with few or no tears and unusual sleepiness or fussiness are frequent signs of dehydration [111]. Children may develop more than one episode of rotavirus disease since neither the vaccine nor natural infection can provide full protective immunity against future infections. Notably, the first infection of a child tends to produce more severe symptoms than recurrent ones [111].

Rotavirus-associated illness is not distinguishable symptomatically from those resulting from other enteric viruses [108]. However, few features distinguish those with RV gastroenteritis from those with other causes of gastroenteritis [112]. People with RV gastroenteritis reportedly manifest more with all three symptoms (fever, vomiting, and diarrhea) and symptoms are more severe compared to illness in people infected by other gastrointestinal viruses [108,109].

## 5. Immunity to Rotavirus

### 5.1. Innate Immune Response

The innate immune response against rotavirus begins with the induction of IFN production, which is mediated by viral dsRNA [113]. After viral penetration of host cells, rotaviral replication is immediately recognized by the host receptors called the retinoic acid-induced gene-1 (RIG-1), Toll-like-receptor-3 (TLR-3), or melanoma differentiation-associated gene 5 MDA-5). Rotavirus nucleic acid is a potent inducer of the host pattern recognition receptor (PRR) machinery which includes the RIG-I, MDA-5, and TLR3 [113].

The absence of 5′-caps is a virus-specific signature that increases the possibility of RV (+) RNAs recognition by RIG-I that enhances IFN expression and an antiviral response [114]. This interaction is followed by the activation of two transcription factors, namely the interferon regulatory factor 3 (IRF3) and the nuclear factor kappa light chain enhancer of activated B cells (NF-κB). The transport of molecules to the nucleus is accompanied by activation of the interferon-stimulated genes (ISGs). During viral replication, NSP1 production can trigger degradation of IRF3, and with the help of rotavirus-dependent and independent mechanisms; the translocation of NF-κβ to the cell nucleus can be blocked. Interestingly, the anti-interferon type I function of the NSP1 protein is different depending on the strain of rotavirus (targeting IRF 3, 5, 7 or TrCP beta) [115]. For instance, human rotaviruses have been shown to rely majorly on the NSP1-mediated degradation of IRF5 and IRF7 to block signaling by IFN-β, whereas NSP1 from rotaviruses of animal origin preferentially targets the IRF 3, 5, and 7, a difference that explains the expanded range of attack exerted by the animal rotaviruses on the IFN-β signaling pathway [115]. In the course of the disease, there is INF transcription and dsRNA-dependent protein kinase (PKR) modulation of more INF generation. This cascade of reactions leading to autocrine production of IFN produces signals that trigger transcription of signal transducers and activators of transcription 1 and 2 (STAT1 and STAT2) and interferon regulatory factor 9 (IRF9). The translocation of these molecules into the cell nucleus will lead to the enhancement of transcription levels of ISG and INF and subsequently, the establishment of an antiviral state for virus clearance and localization to the gut to prevent extraintestinal spread [115,116]. The PRRs mediated activation is characterized by elevated levels of IFN-α, IFN-β, IFN-γ, proinflammatory cytokines and chemokines (TNF-α, IL-6, IL-8, IL-12, MCP-1), in the intestinal mucosa. The IFNs and cytokines from the intestinal epithelial cells and immune cells promote the development of protective immunity through induction of antiviral state, recruitment and activation of immune cells as well as the maturation of dendritic cells (DCs) [116]. The matured DCs become more efficient to connect innate and adaptive arms of the antiviral immune response through priming and activation of T and B cells responses [117]. A fundamental role for TLR-mediated defense against rotavirus is evident from the observation that the absence of MyD88, which is a key convergent adaptor in signaling from the different TLRs culminates in increased viral infectivity, the intensity of diarrheic morbidity, and impaired humoral immunity [118].

Rotavirus infection of a cell triggers 2′-5′-oligoadenylate synthetase (OAS)/RNase-L immune pathway activation. In a reaction cascade elicited by the interplay of viral dsRNA and 2′-5′-oligoadenylate synthetase, the 2′-5′ oligoadenylates are released to cause RNase- L degradation of both viral and cellular RNAs [119]. Recent studies have shown that the VP3 protein of rotavirus synthesized in the cell has a phosphodiesterase activity that antagonizes the deleterious innate immune response through catalytic cleavage of the 2′-5′-phosphodiester bond of the oligoadenylates [120].

Although rotavirus has developed strategies to evade some interferon and NF-κB signaling [121], the innate immune response through the NOD-like receptor (NLR) Nlrp9b inflammasomes represent additional checkpoints used by the host against viral invasion of the intestinal mucosa [121]. According to Zhu et al. [121], conditions that deplete the levels of Nlrp9b or other forms of the inflammasome in the intestine in vivo promote susceptibility to rotavirus replication with consequences such as high viral load, increased viral shedding in stool, and recurrent episodes of diarrhea. Innate immune response via the Nlrp9b inflammasome signaling involves Nlrp9b recognition of short dsRNA sequence of RV, forming of complexes with Asc and caspase-1 and the upregulation of interleukin-18 and gasdermin D expression, which modulate host innate anti-RV defense.

### 5.2. Humoral Immunity

Rotavirus elicits both local intestinal (sIgA) and systemic antibody (IgA and IgG) responses [122]. The immunogenic outer layer proteins (VP7 and VP4) elicit neutralizing IgG and IgA responses, which protect children and adults from disease. The sera from convalescing individuals have also revealed virus-specific antibodies, but which are non-neutralizing against the immunodominant epitopes of RV proteins VP2 and VP6 [122]. The full clinical significance of such non-neutralizing RV-specific antibodies for protection remains to be determined. Specific systemic IgG and IgA at high titers (e.g., >1:200) have been correlated with host protection against RV infection. Similarly, a significant correlation exists between IgA titers and rotavirus vaccine efficacy [123].

Although the small intestine is the primary site for rotavirus infection and replication, reports have shown that viral escape from the gastrointestinal tract thus occurs and this has resulted in antigenemia and genomia associated with systemic and mucosal humoral responses [124]. Homotypic immunity leading to neutralizing antibodies against the major G serotype of the infecting strain is elicited after initial naturally occurring or vaccine-induced rotavirus infection in infants and young children [125]. Subsequent rotavirus infections elicit both homotypic and broader heterotypic (against strains with different G serotypes) antibody responses. Children re-infected by similar strains are significantly more protected than with different G serotypes. The reason suggested is that humoral responses are initially induced against the surface exposed VP7 and VP4 epitopes, while repeated exposure is only required for antibodies elicited against either non-neutralizing, conserved epitopes of the identical proteins or different forms of RV-encoded proteins [70].

Both forms of systemic antibody are correlated with protection against rotavirus infection [126]. However, findings from animal studies and adult volunteers have shown that measurement of local antibodies is better for mucosal surrogates of immune protection against rotavirus illness [127]. Additionally, a recent study by Sinha et al. [128] showed that the circulating antigen-specific antibody-secreting cells (ASCs) that move to lymphoid tissue or specific mucosa site to secrete IgA or IgG antibodies following natural infection or vaccination may also be a good correlate of immune protection against rotavirus in the community, since they appear early and are triggered by all RV strains at high levels during mucosal and systemic infection. However, the results also suggested that RV-specific blood ASCs response, which functions in the homing of plasmablasts to the gut, was short-lasting.

In a recent neutralization study by Caddy et al. [129], high levels of IgG targeting the VP6 (middle capsid particle) of rotavirus were observed, suggesting that the VP6-specific IgG may contribute to the current mechanistic correlates of immune protection. Importantly, the findings of higher efficiency of intracellular neutralization of RVs by the VP6-specific IgG associated with the cytosolic antibody receptor TRIM21 activity as compared to the VP6-specific IgA, confirms VP6-specific IgG protective role during infection and the VP6 as a potential vaccine target.

### 5.3. Cell-Mediated Immunity

In children infected with rotaviruses, the CD4 and CD8 T-cell responses are majorly through Th1 and sometimes Th17 responses. Once activated, proinflammatory cytokines, especially IFN-γ and IL-17 from CD4 and CD8 T-cells, exert an immune-protective response via induction of a direct anti-viral state and recruitment of inflammatory cells capable of viral clearance [130,131]. During rotavirus infection, the regulatory T-cells subpopulations (IL10+ and FOXP3+ regulatory T-cells) are sometimes involved in the suppression of pro-inflammatory immune response to preserve mucosal homeostatic balance in response to rotavirus [132]. Although the B lymphocytes play a major role in the protection against reinfection from the wild-type virus, viral clearance during primary infection is facilitated by the CD8+ T cells [107]. In a lymphoproliferative assay, the decline of rotavirus-specific T-cells after a serologically confirmed rotavirus infection in children and the development of a strong and consistent lymphoproliferative response in healthy adults is suggestive of the vital role T-cells play in viral clearance and protection [133]. The CD4+ T cells provide the necessary signals that assist B and T cells differentiation during infection, with an additional direct anti-rotaviral activity as was demonstrated in recombinant VP6-immunized mice.

The result of chronic infection in mice model with absent T and B cells when challenged with rotavirus highlights the significance of adaptive immunity in the protection against RV disease [134]. The cell-mediated immune response provides resistance against re-infection through the production of cytokines majorly by T-cells and macrophages during the activation and pathogenesis of infectious diseases. This cellular aspect of immune cells secrete cytokines such as IFN-γ and TNF-α that exert antiviral defense through inhibition of virus infection or by modulating a wide range of host immune responses. This response may enable the host to contain or clear the virus as well as protect the host in the acute phase before other aspects of immune responses such as the serum antibody responses comes into play. For instance, the type-1 IFNs (IFN α/β) are elicited to enhance NK cell cytotoxicity and activity, induce MHC 1 expression, upregulate costimulatory molecules on dendritic cells, and promote the expansion of specific memory CD8 + T cell subsets [135].

The findings of strong proliferative T cell responses to RV without an increase in RV antibodies in some young prospectively followed-up children suggest that seroconversion may not always be an exhaustive indicator of early virus exposure as some infections may be missed [130,133]. Therefore, measuring virus-specific T cell responses in infants and small children can complement antibody detection in identifying early exposure to the virus. The fact that passively acquired maternal antibodies do not interfere with T cells results in interpretation showed it may be a useful marker for the early infection [130]. However, they are short-lived and the acute nature of RV infection is such that memory T cells are induced at relatively low frequency. Consequently, small children have less chance of developing circulating memory cells due to the limited exposure history and immature immune system [136]. With advancing age, antibody responses to RV generally remained high, leading credence to the current consensus that seroconversion is a better marker of protection against rotavirus [137].

## 6. Laboratory Diagnosis of Rotavirus Infection

Laboratory diagnosis of rotavirus infection involves testing of fresh, whole stool samples or rectal swabs from diarrheic patients for the presence of the virus, virus-specific antigen, or RNA [60]. Direct detection of rotavirus involves the use of electron microscopy (EM), a sensitive and highly specific method. A recent modification utilizing magnetic microparticles functionalized with monoclonal antibodies enhanced the ability to capture, concentrate, separate, and detect infectious rotavirus particles in clinical samples [138]. However, the method of EM is expensive, requires highly trained personnel, and is labor-intensive for the routine detection of rotavirus in large numbers of specimens [139]. Commercially available antigen detection kits (ELISA, immunochromatography, or latex agglutination) are primarily used for rotavirus diagnosis. The latex agglutination technique is rapid and simple to carry out without sophisticated equipment, making it useful in disease outbreak detection especially in resource-poor settings where means for rotavirus recognition are in short supply [140]. Although, the ELISA-based technique is the most widely explored antigen screening platform due to its high sensitivity, specificity, and adaptability to a large sample volume of samples in the 96-well plate [60]. Growing rotavirus in cell culture helps to confirm viral viability and also improves the molecular detection of the virus, which may be present in very low concentrations in the clinical or environmental samples [39]. Although cell-culture-based methods are highly sensitive, they are laborious and expensive. It is time-consuming, highly prone to contamination, and is often not requested for clinical diagnosis.

Polymerase chain reaction (PCR)-based technique (e.g., reverse transcription (RT)-PCR, qPCR, real-time PCR), which detects RNA in the clinical sample, is a more sensitive method than antigen detection platform but, to date, it remains primarily a research tool [60]. The sequencing of VP7, VP4, and other genome segments is required for genotyping circulating rotavirus strains. Conventional sequencing techniques have the disadvantages of being labor-intensive, low throughput, and costly [141]. Newer RT-qPCR assays, especially TaqMan^®^ assays, have been used to overcome the challenges of conventional RT-PCR and sequencing. Compared with other methods, real-time quantitative PCR has advantages of increased specificity, sensitivity, genotyping, ability to multiplex, high throughput sample processing, faster turnaround time, and quantitative accuracy [141]. For complete characterization of RV genome and identification of unusual genotype constellations, whole genome analysis has recently been recommended by the RV classification working groups [20]. However, the method is yet to be employed routinely due to the increased resources required.

## 7. Epidemiology and Molecular Diversity

### 7.1. Morbidity and Mortality in Children

Rotavirus is the leading cause of diarrheal morbidity and mortality in young children worldwide. The infection is generally acute and severe with a high rate of dehydration often needing hospitalization. Dehydration, if not treated early, may lead to death, as it is commonly seen in developing countries [13]. Generally, most children experience an episode of RV gastroenteritis (RVGE) before their fifth year birthday, with one in every five of them visiting a health facility, one in every 65 cases necessitating hospitalization, and approximately one in every 293 cases eventually having a fatal outcome [142,143].

Rotavirus-induced diarrhea was responsible for the annual death of about 527,000 children ≤5 years across the globe before rotavirus vaccine use. This rate accounted for approximately 40% of all diarrheal deaths and 5% of all deaths among the under-five children [60,144]. According to a report, greater than 90% of RVGE deaths noted in 2013 occurred in 72 low and middle-income countries [143]. Implementation of RV vaccination in national immunization programs (NIPs) reduced the RV disease burden substantially. Post-vaccination studies documented a death rate attributable to rotavirus diarrhea among ≤5 at approximately 215,000 per annum [1]. However, the epidemiological distributions of disease burden vary remarkably across various geographical settings [60]. For instance, in Europe, acute gastroenteritis cases due to RVA account for 75,000–150,000 infantile hospitalization. In Spain, the annual incidence of acute gastroenteritis associated with RVA in primary care ranges between 15.4 and 19.5 cases per 1000 children up to 5 years and 20 cases per 1000 children up to 3 years [145]. In the Eastern Mediterranean region, the annual morbidity rates ranged from 0 to 112/100,000 with an average mortality rate of 39/10,000 per year [146]. Generally, higher mortality rates due to RVGE were noted in the low-income countries (e.g., Afghanistan, Pakistan, Sudan, Yemen, and Somalia) compared with countries where the per capita income was high (e.g., Saudi Arabia and Kuwait). However, the overall hospital and health center visits due to RVGE among under-five were similar in both high- and low-income WHO-EMRO countries [143,146]. A recent meta-analysis finding of Ardura-Garcia et al. [147] among under-five year children in highly developed countries showed rotavirus is responsible for 21% (95% CI 16–26%) acute gastroenteritis cases necessitating primary health care utilization, 32% (25–38%) visits to the emergency department; 41% (36–47%) hospitalization, 29% (25–34%) nosocomial infections and 12% (8–18%) diarrheal deaths.

There are three major immunologic groups of rotavirus with distinct epidemiologic distribution patterns. The group A Rotavirus (RVA), which accounts for >90% of rotavirus gastroenteritis cases in humans, is endemically distributed worldwide [19]. RVA has caused significant numbers of outbreaks among hospitalized infants, young children at family homes or daycare centers as well as the elderly care homes [27,30,148]. Large outbreaks of RVA attributable gastroenteritis have been reported in Brazil [149], Nicaragua [150], and Botswana [151]. Rotavirus B, commonly referred to as adult diarrhea rotavirus or ADRV, is responsible for the sporadic and sometimes epidemic cases of the outbreak in humans [152].

Globally, human RVAs G-genotypes designated G1-G4, G9, and G12, as well as P-genotypes P[4], P[6], and P[8], predominate [153]. Molecular epidemiological studies across the globe have identified more than 60 G/P combinations circulating in human populations. The G/P genotypes combinations frequently implicated in human infections worldwide are G1P[8], G2P[4], G3P[8], G4P[8], G9P[8], and G12P[8] [56,154]. These globally predominant strains exhibit temporal and regional variations, which influence the diarrheic episodes in some RV seasons. For instance, in Africa, these strains are responsible for nearly 63% of all RV infections, whereas in Europe, it accounts for >90% [55,56]. In a single season, the majority of the prevalent strains may co-circulate, thereby increasing the likelihood for genetic diversity by the mechanism of genome reassortment [155]. In the developing countries where factors such as overcrowding, sharing of a common source of water, and living space by domestic animals and humans are high, the uncommon human G/P type combinations are frequently reported due to the increased chances of interspecies transmission of rotaviruses and reassortment events [56]. Thus, the unusual rotavirus genotypes such as G1P[4], G2P[8], G9P[4], G12P[4], G8P[6], G8P[8], and G12P[6] have acquired greater epidemiological relevance in some rural areas of Africa, Asia and South America [156,157].

### 7.2. Age and Sex Incidence Distribution

In developing countries, the attack rate is very high among children aged 6 to 12 months whereas children of 12 to 14 months were predominantly infected in developed countries [158,159]. About 38% of children develop protective immunity to the virus after the first natural challenge with rotavirus, 77% of them are protected from acute rotavirus-induced diarrhea while 87% do not come down with severe cases [60]. Rotavirus infections in adults have occurred among the military population, hospital personnel, immunocompromised patients, elderly, travelers to developing countries, and parents in homes of infected infants. About one in every three adult infections is clinically inapparent, although reinfection in both children and adults does occur [30,60]. Boys infected with rotavirus are more likely than girls to be admitted to the hospital [160]. The reason for this difference has not yet been proven. The period of highest susceptibility usually corresponds with the decline of maternally acquired immune factors that often wanes after about 5 months. Consequently, susceptibility to rotaviral disease continues for a lifetime. Though the majority of severe cases occur at the infants’ first infection [60].

### 7.3. Seasonal Patterns of Infection

Rotavirus infections occur primarily during cool, dry seasons [161]. The seasonality of rotavirus infections differs from one region to the other. It is regarded as “winter diarrhea” in some parts of the world where the majority of the cases are seen in the winter season [162,163]. In the tropics, rotavirus infection occurs all year-round, although with fluctuations characterized by peaks and valleys, whereas in the temperate regions incidence is almost zero in certain months, but peaks during the fall and winter. The generally low climatic variability in tropical areas may not be sufficient to cause a significant change in disease incidence. In Africa for example, rotavirus infections occur all year round in all most every country, with different peaks during the dry months compared to the wet periods. Only a few countries in the continents with variable climatological variables may have a different pattern of disease occurrence. In South Asia, the peak of rotavirus infection occurs in the colder, drier months of the year [32]. As survival of infective rotavirus is favored in cooler conditions with low relative humidity, it has been hypothesized that a relative drop in humidity and rainfall combined with the drying of soils might increase the aerial transport of dried, contaminated fecal material [162].

Researches have shown that with a 1 °C increase in the mean temperature in the tropical region, the rotavirus incidence decreases by 10%. Additionally, with a 1 cm increase in mean monthly rainfall, the incidence of rotavirus decrease by 1%. The seasonal pattern may also be influenced by socio-demographic factors [32]. This observation corroborated earlier reports that identified income level as a stronger predictor of seasonality than latitude or geographic region. The study of Patel et al. [162] identified level of country development as a stronger predictor of the seasonal intensity of rotavirus disease compared to latitude or the geographical location of each poorer country, particularly those in Africa, Asia, and South America that had lesser seasonal variation in disease than the more developed countries from Europe, North America, and Oceania, even after taking into account local climate and geographical location. Generally, tropical countries are less developed than those in temperate regions, thus increased opportunities for high transmission rates and high birth cohort behaviors in those poor countries are more likely and could be the reason for relative lack of seasonality in these countries [164]. The peak rotavirus activity in the US begins in the Southwest in autumn (October–December) and ends in the Northeast during spring (March–May) [163]. An epidemiological study of RV transmission dynamics in the US showed that demographic parameters such as spatiotemporal disparity in birth rate could drive the differences in seasonality of RV diarrhea in different geographical settings [165]. Thus, in the developing countries where birth rates are high, the seasonality of RV diarrhea is less marked as a significant number of new susceptible children are introduced into the population all year round [165].

### 7.4. Nosocomially-Acquired Infection

Acute gastroenteritis related to RV is defined as nosocomial when the symptoms appear at or after 48 h of admission in the hospital to 72 h after hospital discharge. Reports of several studies have shown that between 15–30% of cases of nosocomial RV infection have occurred after hospital discharge with an addition of 0.8–1.0/100 cases being for the seasonal incidence in infants and toddlers [166]. Generally, viruses are the most recognized agents for nosocomial disease in the pediatric ward with nosocomial diarrhea resulting in 91–94% of all cases, 65–90% of pediatric hospital-acquired infections, and 31–87% of cases attributable to RV [167]. The introduction of RV in the pediatric wards mostly results from hospitalized children who had acquired RV from the community, particularly after their stay in the emergency room before being hospitalized. However, RV symptoms resulting from community-acquired and nosocomial RV infections are not distinguishable. Viral excretions usually begin shortly before the onset of disease symptoms. Even after the resolution of diarrheic symptoms, the individual may continue viral shedding for as long as 57 days. Individuals are usually infectious within the first 2 weeks, though it could be extended in immunocompromised patients. People not showing symptoms of RV accounts for 18–39% of all nosocomial RV cases in which preponderance of cases are seen in neonates and children <3 months old. The social impact is low but broad, essentially impacting a month of a family’s life, without sequelae. Studies have found that out-of-pocket costs (rehydration therapy, non-prescription drugs, diapers, phone calls, and transport) and time lost from work are considerable for the families of affected children, even for cases of low severity [168].

## 8. Prevention and Control

### 8.1. Vaccination

The control of rotavirus-attributable diarrheal diseases currently relies on the use of live attenuated oral rotavirus vaccines especially in countries where the mortality rates are high [22]. RotaTeq (RV5) and Rotarix (RV1) are the most widely used vaccines for the prevention of rotavirus infection globally since WHO pre-qualification in 2008 and 2009, respectively [169] (Table 2). Rotarix is an oral monovalent vaccine consisting of a live-attenuated human rotavirus G1P[8] genotype. The breakthrough vaccine virus was derived from the stool of a <12 months-old baby with natural RV infection, and viral attenuation was achieved through cell culture passages [170].

RV1 is a product of GlaxoSmithKline Biologicals, Belgium, and was launched into the market in 2006 [78]. Since then, the use of RV1 is extensively characterized by post-marketing surveillance studies in different settings to establish safety, effectiveness, and impact [171,172]. Such data are especially needed by countries intending to switch from Gavi support to self-financing, as they serve as an evidence-based rationale for sustained support of rotavirus vaccination [169]. The pooled efficacy data from the developed countries have shown that RV1 prevents 82% of severe diarrhea cases attributed to RV and approximately 37% of severe all-cause childhood diarrhea. In the developing countries, on the other hand, RV1 prevents only 35% of severe rotavirus-attributable diarrhea cases, which account for 17% of all-cause of severe childhood diarrhea episodes [171]. RotaTeq is an oral pentavalent live attenuated reassorted bovine-human rotavirus vaccine containing four common human VP7 (G) types (G1, G2, G3, and G4) and one common human VP4 (P) type (P[8]). RotaTeq was developed by Merck and Co. Inc., USA, and launched in the market at the same time as RV1 [171]. In developed countries with reportedly low mortality, RV5 has been shown to prevent 82% of severe rotavirus-associated childhood diarrhea. Similar to R1, the effectiveness is reduced in developing countries with notable high birth cohorts as it only prevents 41% of severe rotavirus-associated childhood diarrhea and approximately 15% of severe all-cause of diarrhea episodes [171]. One rationale for rotavirus vaccination is that it does not only elicit an immune response to the serotype in the vaccine, but also the heterologous serotypes [172]. RotaTeq^®^ is administered in 3 doses at ages 2 months, 4 months, and 6 months while Rotarix^®^ is in 2 doses at ages 2 months and 4 months [60].

The World Health Organization in 2018 prequalified two additional vaccines namely; ROTAVAC^®^ (Bharat Biotec of Hyderabad, India) and ROTASIIL^®^ (Serum Institute of India, India) (Table 2). Rotavac is a monovalent vaccine containing a live-attenuated wild-type reassortant G9P[11] rotavirus strain whereas the Rotasiil is a pentavalent vaccine containing a lyophilized preparation from reassortant human-bovine rotavirus G1–G4 and G9 strains [60]. Meta-analysis findings from a pooled efficacy studies have shown that Rotavac prevents 54% of severe rotavirus-associated diarrhea cases in India, which account for a 16% reduction of all cases of severe diarrhea episodes [171]. Both Rotavac and Rotasiil have been licensed internationally and have since been introduced by India. Rotavac is currently in use in Palestine and some African countries [4,78]. Elsewhere in the World, as of the end of 2018, 106 countries have included Rotarix or RotaTeq rotavirus vaccines in their national childhood immunization programs [78].

Two vaccines namely; Rotavin-M1 (POLYVAC, Thành phố Hà Nội, Vietnam) and Lanzhou lamb (Lanzhou Institute of biological product, China) are currently been licensed nationally in Vietnam and India, respectively, to promote affordability and availability of rotavirus vaccination. Both vaccines are yet to receive prequalification by WHO and their coverage is limited [60,78]. Although these two vaccines are promising, large efficacy studies and impact survey data on both vaccines are currently unavailable and are warranted to provide a clue on their performance on a larger scale [170]. The license of Rotavin-M1 in Vietnam in 2012 was based on the tolerability outcome as well as the immunogenicity rate, which was estimated at 73% (Ig)A seroconversion in a trial of Vietnamese children [173]. Rotavin-M1 contains the G1P[8] strain and the frozen preparation is administered orally with a two-dose schedule at ages 2 and 4 months [174]. The Lanzhou lamb contains G10P[15] rotavirus genotype and is given in a single dose followed by annual boosters for children aged between 2 months and 3 years [175].

Before vaccine introduction in the US, RV was responsible for about 2.7 million diarrheal cases each year in which >95% of the children were infected before their fifth birthday. After the execution of the rotavirus vaccination, about 280,000 hospital visits, 62,000 visits to the emergency department, and 45,000 hospitalizations were averted annually [176]. The post-vaccination era also witnessed indirect protection of unvaccinated age groups population and an overall decline in healthcare costs [177]. The recent report of the 12th African Rotavirus Symposium showed that approximately 40% rates reduction in hospital admission of under 5-year-olds children with acute gastroenteritis was observed between 2006 and 2018 following the introduction of RV vaccine in WHO-coordinated African region comprising 33 Member States [178]. A global estimate showed that rotavirus infection accounted for 453,000 deaths (95% CI, 420,000–494,000) in children <5 years and 37% of deaths due to childhood diarrhea before rotavirus vaccination was introduced [142]. Since the WHO recommendation for the inclusion of the rotavirus vaccine in all national childhood immunization programs over a decade ago, rotavirus-associated diarrheal morbidity and mortality have declined substantially across the globe [1,170]. Although, due to inadequate rotavirus vaccine coverage and high birth cohorts in some countries, the virus was still responsible for approximately 128,500 deaths (95% CI, 104,500–155,600) among children <5 years globally in 2016 with greater attributable percentage death rates in low- and middle-income countries [1].

The current advances in reverse genetics system for RVs involving the use of an entirely plasmid-based platform has been regarded as a breakthrough and a key technological advancement over the more tedious helper virus-dependent reverse genetics techniques developed for RVs [179]. In a recent study, a plasmid-based RV reverse genetic system was successfully employed to generate both NSP3 and a fluorescent reporter protein by replacing the open reading frame (ORF) of segment 7 of RV dsRNA with an ORF encoding NSP3 that is fused to a fluorescent reporter protein [180]. The successful manipulation of RV genomes without affecting the reverse genetic replication in vitro and the generation of two heterologous proteins shows the potential use of rotaviruses as an expression vector system for the delivery of bivalent vaccines. Further, the use of recombinant rotaviruses as an expression vector of the highly immunogenic protein domain of the SARS-CoV-2 [181], is suggestive of broader usefulness when fully developed. Studies have shown that the reverse genetic system not only permits the regulation of the experimental conditions, but also the preferred combination of several RV gene segments with concurrent mutations and the production of mutants with a reduced interferon response [179,181]. All these features of the reverse genetic system-based approach may be beneficial in advancing the development of next-generation rotavirus vaccines.

#### Factors Influencing RV Vaccine Efficacy and Effectiveness in Poor Socioeconomic Settings

Generally, the efficacy and effectiveness of the oral rotavirus vaccine are higher in developed countries compared to the developing nations where RV mortality is higher [171] (Table 3).

**Figure 3:**
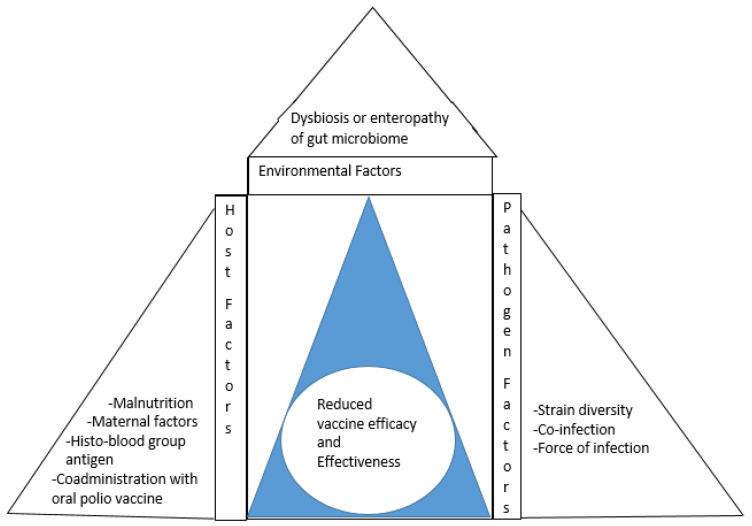
Epidemiological triad of factors impacting oral rotavirus vaccine performance.

The differences in vaccine effectiveness have also been observed in connection with oral polio and typhoid vaccines [191]. Several hosts, pathogen, and environmental factors have been suggested as the driving force for the discrepancies in vaccine performance between the two socioeconomic settings [7,192]. Other barriers to achieving the full potential of the vaccine for global rotavirus disease prevention include age restrictions on vaccine use, concern for safety attributed to intussusception and reversion to virulence in malnourished or immunosuppressed individuals due to the live attenuated nature of the vaccine virus, inhibitory effect of the maternally acquired antibodies against RV, and the cold chain storage requirement, which is difficult to maintain in low resource settings [67] (Figure 3).

**I. Host associated factors**

Globally, malnutrition is responsible for approximately 45% of deaths among children younger than 5-years old, with a preponderance of cases in low- and middle-income countries [27]. Malnutrition related either to protein-calorie (kwashiorkor) or essential micronutrients (vitamins and mineral elements) is posing a serious concern for global health, as it remains the leading cause of immune deficiency, with attendant effects on the intestinal microbiota balance and immune responses to oral vaccines [193,194]. Experimental findings from animal models have shown that both immunoregulatory responses and protective efficacy of oral live attenuated human monovalent and pentavalent RV vaccines were affected by vitamin-associated-deficiency [192,195]. Retinoic acid, a derivative of vitamin A, helps in the activation of gut dendritic cells and upregulation of gut homing receptors such as CCR9 and α4β7 on vaccine-induced B and T cells [196]. In developing countries, nutrients deprivation (infant formula or breast milk) attributed to prevalent factors such as infection, poor sanitation, and poverty often predisposes infants to malnourishment [106]. Malnutrition has been shown to affect both the innate and adaptive immune responses to RV infection. Consequently, reduced protection against RV diarrhea has been observed in different studies among malnourished children post-vaccination challenge [197,198]. In a gnotobiotic neonatal pig model of childhood malnutrition, a decrease in RV-specific IgA and IgG class in serum and intestinal tissues, as well as RV-specific IgG and IgA antibody-secreting cells in the blood and intestinal tissues, has been observed following RV vaccination when compared with nutrient sufficient animal. The findings that malnutrition is associated with impaired mucosal and systemic RV antibody responses post-vaccine challenge and infection suggest it can reduce both the protective efficacy and effectiveness of oral RV vaccines in children in developing countries [199]. In addition to B-cell impairment, protein deficiency has been shown to impair many aspects of innate, adaptive T-cell, and cytokine immune responses that resulted in decreased protective efficacy of an oral RV vaccine in a microbiota humanized animal model. Furthermore, malnutrition associated with impaired T-cell immunity has been shown to exacerbate disease severity and also prolong virus shedding following challenges with virulent RV [200]. This, in part, may explain why the high rate of RV-associated diarrheal mortality still exists in impoverished countries despite the availability of oral vaccines.

Human breast milk has been shown to contain antibodies and other immunological factors that can inhibit RV replication in vitro and also reduce the immunogenicity of the virus components of the oral vaccine, especially when babies are breastfed close to the time vaccine is administered [201,202]. This has been corroborated in observational trials, where higher levels of rotavirus-specific IgA antibody in breast milk were akin to failed seroconversion [203]. On the contrary, findings from several clinical trials have shown that withholding or restriction of breastfeeding at the time of vaccination did not enhance the rate of IgA immune response to oral RV vaccines in children [204,205,206]. A higher rate of IgA seroconversion had been observed in infants immediately breastfed than those withheld from a feeding [206]. More recently, emerging information from clinical trials showed higher levels of rotavirus-specific IgG antibodies acquired through the placenta rather than the antibodies ingested through breastfeeding were significantly associated with reduced vaccine-elicited immune responses in infants [203,207]. Nevertheless, the general findings of higher levels of rotavirus-specific antibodies in both maternal serum and breast milk in the low and middle-income countries compared to high-income countries are suggestive of greater potential for inhibition [202,203]. While the clinical relevance of rotavirus-specific antibodies in maternal sera and breast milk with oral vaccine immunogenicity and protection from rotavirus disease is still being investigated across different socio-economic settings, also including studies featuring on the understanding of the mechanisms of inhibition may further shed light on their impact on vaccine performance.

The simultaneous administration of some vaccines with the RV vaccine has been shown to impart on RV vaccine effectiveness. For instance, the concurrent administration of the RV vaccine with oral polio vaccine (OPV) in low-income countries has been shown to inhibit immunological response to RV vaccine [169,208,209]. In high-income countries where OPV is no longer in use, the interference of the OPV causing lower titers of antibody to the rotavirus vaccine is avoided.

**II. Pathogen associated factors**

Certain intrinsic factors of RV such as the force of infection (FOI), enteric coinfections, and genetic diversity have been suggested as driving factors for the decreased vaccine effectiveness in low- and middle-income countries compared to high-income countries [207]. The force of infection refers to the rate of infection of susceptible individuals in a population per unit time and is influenced by pathogen transmission intensity, susceptibility of the host to infection and disease, and the level of protective immunity resulting from vaccination across settings [210].

The FoI of RV displays significant global and local heterogeneity within countries. For instance, a mixed model study of the incidence of rotavirus infection in children from two low-resource settings in India and Malawi showed that children in the former were exposed to rotavirus at a much younger age than those in the latter [211]. In low resource settings, prevalent factors such as poor sanitation and hygiene, inadequate water supply, and low vaccine coverage have been shown to significantly contribute to higher RV transmission intensity and the early peak of RV incidence compared to their counterparts in high resource settings [7]. The early and multiple exposures to rotavirus orchestrated by the greater FOI can cause the production of an active immune response similar to maternal antibodies, which may impact vaccine response in infants [7,202]. Understanding and modifying the FoI between two socio-economic settings may provide the most direct, proximate, and actionable interventions such as providing an additional dose of vaccine [212], considering a neonatal dose schedule [213], or delaying a vaccine schedule [212] to improve or sustain vaccine response.

Children from low resource-poor settings have generally shown much higher rates of enteric co-infections than those from high-income regions. For instance, a 77% enteric co-infection rate reported in a Ghanaian study was 10-fold higher than the rate observed in children living in a French country [214,215]. Findings from several multicenter studies employing broad molecular-based testing have indicated higher enteric coinfection with RV across developing countries in Africa and Asia as compared to high resource settings [216,217,218]. The association of enteric coinfection with prolonged diarrheal episodes and accentuation of RV disease severity shows that the comorbid condition can affect the protective efficacy and effectiveness of the rotavirus vaccine [219].

**III. Environmental associated factors**

The microbiome of the gut supports host defense and homeostasis in recovery from gastrointestinal infections [220]. The stress-induced by both biotic and abiotic factors reduces the functionality of the microbiome and lowers the production of metabolites required by the host [221]. The development of the microbiome begins soon after birth and matures by 2 years of age. The stages of development are characterized by constant changes in microbial structure and composition, which are often influenced by environmental factors such as delivery mode, breastfeeding status, nutrition, probiotic/prebiotic, and antibiotics [192,222]. In resource-poor settings, fecal contamination caused by poor water, sanitation, and hygiene is widespread, and this has been shown to contribute substantially to intestinal pathology which in turn reduces the immunogenicity of oral rotavirus vaccine [191]. Additionally, the poor state of sanitation creates multiple chances for interspecies transmission and reassortment events, which favors the emergence of atypical or novel strains with potential for impaired vaccine efficacy. Generally, altered gut microbiota composition has been shown to affects direct RV–microbiota interactions leading to an inefficient vaccine virus strain replication in the intestinal tract and decreased immunogenicity of oral live attenuated vaccines [192,223]. In resource-limited settings, the alteration of gut microbiota or the use of antibiotics that perturbs the gut microbiota balance have been reported in association with reduced immunogenicity of rotavirus and other oral vaccines [224,225]. A study by Srivastava et al. [193] investigated the interrelationship between the host microbiota, nutrition, and human RV vaccine by challenging the neonatal gnotobiotic pig’s model that was fed with a protein-deficient or sufficient diet with oral RV vaccine. In the former, alteration of gut microbiota composition was correlated with the poor immune response to the vaccine whereas, in the latter, a high level of rotavirus vaccine efficacy attributed to the intact gut microbial structure was observed.

### 8.2. Nonvaccine Approaches

#### 8.2.1. Good Hygiene

Although comparable incidences of rotavirus disease between the developed and developing countries have shown that the disease cannot be exclusively controlled with hygienic measures such as well personal, food, and environmental hygiene, a further improvement may help in breaking transmission and severe episodes of diarrhea. In ensuring hand hygiene, regular washing of hands with liquid soap and water and then rubbing for at least 20 s should be practiced before handling food or eating and after using the toilet. It is also recommended that food handlers should adopt all the food safety procedures to minimize the chances of contamination [226].

#### 8.2.2. Breastfeeding

The WHO guidelines of pediatric diarrhea management include continued breastfeeding to reduce the length and severity of diarrhea. Breast milk contains bioactive components such as antibodies, antioxidants, nutrients, and hormones, which protect a child from specific pathogens or families of pathogens or confer mucosal immunity to the infant [227]. Several studies have documented the protective role of breastfeeding against childhood rotavirus infection or reduction in the diarrheic severity, while some others held the opposite views. Studies by Shumetie et al. [228] and Krawczyk et al. [229] posited that exclusive breastfeeding throughout the first 6 months of life significantly prevents rotavirus diarrhea. According to Shumetie et al. [228], children not exclusively breastfed were about 3-fold more likely to have RV diarrhea. Particularly, a significant reduction in systemic manifestations of RV-antigenemia/RV-RNAemia rate had been observed in breast-fed infants and children compared to the non-breastfed category [230]. These observations contradict previous studies, which indicated a lack of significant correlation between rotavirus diarrhea and breastfeeding [231,232] or no protective effect on viral diarrheal morbidity [159].

#### 8.2.3. Probioses

Probiotics are a group of live microorganisms which, when sufficient amounts are ingested, have the potential to confer a health benefit on the recipients [233]. The organisms such as lactobacillus, Saccharomyces, Streptococcus, Bacillus, Bifidobacterium, and Enterococcus, which are commonly consumed through fermented food items such as milk, cheese, yogurt, and cereal beverages, are noted for their ability to resist gastric acidity and bile juice and to adhere to the epithelial lining of the gut [234]. Their beneficial role as alternatives for treatment and/or alleviating the severity of gastroenteritis caused by rotavirus infections has been attributed to the direct antimicrobial effects through the inhibition of colonization and growth of pathogens, promotion of the damaged mucosal epithelial barrier function, and modulation of both the innate and cell-mediated immunity [235].

The mechanism by which probiotic exerts anti-Rv is currently been investigated. Competition for cellular receptor sites between probiotic bacteria and RV has been suggested in association with the inhibitory effect of probiotics on RV pathogenesis [107]. In vitro and in vivo studies have linked the anti-Rv activity of probiotics to the production of antimicrobial substances (bacteriocins, short-chain fatty acids, lactic acid, H_2_O_2_, nitric oxide, etc.), induction of mucin secretion by mucosal epithelial cells as well as the activation of local adaptive through specific IgA response and innate immune responses [107,236]. For instance, a randomized clinical trial in India confirmed the beneficial roles of probiotics and zinc supplementation on the immune response to RV vaccine among four groups of infants. In the study, the first group received probiotics (*Lactobacillus rhamnosus* GG) and oral zinc (5 mg daily), the second group received probiotics only with zinc placebo, the third group received probiotic placebo with zinc only while the last group received probiotic placebo and zinc placebo. All groups received the intervention a week before the commencement of the Rotarix vaccine series which span for 6 weeks after the second dose [237]. Although, neither zinc nor probiotic significantly increased the rate of RV IgA seroconversion, the probiotic administration impacted a certain degree of health benefits as a 7.5% increase in RV-IgA seroconversion was observed in all infants who received probiotic against those who did not (97.5% CI −1.4–16.2).

Probiotics comprising Lactobacillus and Bifidobacteria species cause the release of signals that trigger the suppression of inflammatory cytokines production when recognized by Toll-like receptors 2 (TLR-2) on dendritic cells. This further promotes a cascade of a reaction involving activation of MAPK (mitogen-activated protein kinase) and NF-κB (Nuclear factor Kappa light chain enhancer of activated cells) pathways, thereby reducing the permeability of tight junctions of intestinal epithelial [238]. The pili of bacterial probiotics such as Lactobacillus rhamnosus interacts with the macrophages to upregulate IL-10 production and concomitantly decrease that of IL-6 following their internalization by macrophages [239].

Probiotics are generally believed to be safe as the microbial compositions are major members of the normal microbiota in humans or animals [233]. Moreover, they only transiently persist in the human intestine after oral administration [240]. Reports from pooled efficacy studies have shown that probiotics safely exert a positive effect in reducing the duration of acute pediatric diarrhea [240]. Though probiotics mechanism against RV is not well defined yet, its safety is a feature that had recently attracted great interest from pediatricians and food microbiologists in their potential use either alone or in combination with the conventional treatment modalities for managing rotavirus diarrhea in infants [241].

#### 8.2.4. Antiviral Drugs

There are currently no approved antiviral drugs for the treatment of rotavirus infections. Although, there have been a lot of studies demonstrating the anti-rotavirus activity of some drugs. For example, gemcitabine, a potent anti-cancer drug, has been shown to inhibit rotavirus through the alteration of pyrimidine nucleotide biosynthesis pathway [242], 2′-*C*-methyl nucleosides inhibit the viral polymerase [243], racecadotril (an intestinal encephalinase inhibitor) suppresses the secretion of water and electrolytes into the gut that is activated after RV infection [244], and Nitazoxanide targets viral morphogenesis to cause inhibition of viroplasm formation [245] (Table 4). Dycke et al. [244] investigated antirotavirus activity of four 2′-*C*-methyl nucleosides comprising 2′-*C*-methylcytosine (2CMC), 2′-*C*-methyladenosine (2CMA), 2′-*C*-methylguanosine (2CMG), and 7-deaza-2′-*C*-methyladenosine (7DMA) using cell culture and animal mouse model. All the four nucleosides completely inhibited rotavirus-induced cytopathogenic changes in vitro and potently reduced viral replication in a mouse model. Chen et al. [246], in a similar cell culture study, demonstrated the robust anti-RV activity of Ziyuglycoside II. In a mouse model, Ziyuglycoside II substantially reduced the viral RNA copy in a dose- and time-dependent manner. Furthermore, the inhibition of Toll-like receptor 4 (TLR4)/nuclear factor kappa-B (NF-κB) signaling pathway was shown to be associated with improvement of diarrheic symptoms and disease severity. Naturally, TLR4 is a pattern recognition receptor for lipopolysaccharide found in both the cell membrane and the cytoplasm. LPS interaction with TLR4 triggers the activation of downstream NF-κB signaling pathways to cause inflammatory response [247]. This activation was blocked in the presence of Ziyuglycoside II.

Brequinar, an inhibitor of mitochondrial dihydroorotate dehydrogenase, has recently been shown to exert a strong inhibition on RV replication through interference with the pyrimidine biosynthesis pathway [248]. Brequinar produced rotavirus inhibitory ID50 dose of 0.05 uM and a very high specificity index (>104) in a cell against an extract cytotoxic CC50 dose of 1613 uM.

Resveratrol, a potent bioflavonoid compound and a major constituent component of biological matter identified in plants and fruits, was recently shown to be a strong inhibitor of viral protein expression and genomic RNA synthesis in vitro and in vivo through antagonism of downstream HSP90 expression needed for viral entry, morphogenesis, nuclear import, transcriptional activation, and replication. In vivo studies in mice showed that anti-rotavirus activity of Resveratrol could further be attributed to its ability to inhibit cellular MEK/ERK kinase signaling pathway, thereby blocking the virus from utilizing host cell signaling cascades or molecules to their replication and survival advantage [249]. Resveratrol commonly found in grapes, nuts, white hellebore, berries, and red wine showed to be a highly promising anti-rotavirus drug nevertheless, its safety profiles in clinical trials need to be investigated. Few of the newly discovered potential anti-rotavirus drugs are been tried in humans for their efficacy and safety. Racecadotril treatment of diarrheic patients is beneficial in reducing the severity of acute diarrhea at 48 h post-treatment without any associated adverse effects, although the drug did not significantly reduce the proportion of diarrheic patients 5 days post-treatment [244]. Meta-analysis findings from seven pooled clinical trials showed that racecadotril treatment effectively reduced the duration of illness and stool output in children with acute diarrhea compared to the placebo or absence of any intervention [250]. Nitazoxanide, an oral synthetic anti-parasitic agent, was recently evaluated for its therapeutic efficacy and safety among diarrheic children with acute rotavirus gastroenteritis from developing countries. The nitazoxanide syrup doses of 100mg in 12–47 months and 200 mg in ≥4 yrs administered twice per day for three consecutive days resulted in a significant decline in the median duration of diarrhea episodes and hospitalization without any undesirable effects [251]. However, the treatment does not produce a significant effect on the median duration of fever or vomiting, which suggests the need for further improvement.

Eichwald et al. [252] showed that a small molecule (ML-60218) regarded as an RNA polymerase III inhibitor specifically disrupts the viroplasms assembly and the formation of the VP6 structure of RV in a dose-dependent manner. In another study, genipin found in the fruit (*Gardenia jasminoides)* was shown to inhibit both the early and late stages of RV replication [253]. The additional findings of downregulated pro-inflammatory cytokines expression from the infected cell [253], suggest genipin could be a potential natural preventive and therapeutic agent against RV infection and its complications.

Currently, the management of rotavirus patients relies on the use of oral rehydration solution to replace the fluids and body electrolytes lost in stool and vomit [254]. This is normally supplemented with zinc tablets to help restore the damaged mucosal epithelial lining. The antibodies to rotavirus found in human or bovine colostrum and human serum immunoglobulin are beneficial in reducing or preventing rotavirus diarrhea, though these are yet to be adopted in routine practice [60]. As there is no proven therapeutic intervention yet for the virus and severe diarrhea it causes, there is the need to give priority attention to the development and improvement of the aforementioned potential anti-rotavirus drugs. The realization of the objectives of potent anti-rotavirus and their combination with the current vaccination efforts could synergistically reduce the global burden of the disease and many needless deaths.

## 9. Conclusions and Perspective

Rotavirus causes acute dehydrating diarrhea associated with high global mortality in particular among under five-year children. The introduction and expanded use of the two oral attenuated rotavirus vaccines have already contributed to reductions in rotavirus-attributable child death and hospitalization. Although rotavirus vaccination is generally acclaimed to be effective in reducing the global impact of diarrheal disease, its underperformance in low resource countries in Africa and Asia, where the mortality rate is highest and opportunities for infection are wide-ranging, calls for an actionable intervention. To overcome this challenge, a multifaceted approach is crucial that can address the various factors that impact ORV performance between socioeconomic settings including the probable need for next-generation vaccines by policy makers. Low vaccine coverage, vaccine-induced selective pressure, rapid rate of viral evolution, and increased opportunities for interspecies transmission can affect the success rate accrued to current vaccination strategies. Following exposure, virus competition for interaction with the cellular receptor (entry-stage) or cellular machinery (after entry) needed for completion of all stages of the replication cycle is a naturally occurring physiological process that cannot be changed by any simple approach. Therefore, a multi-barrier measure to prevent rotavirus infection must be strengthened through improvement in vaccination coverage, water and sanitation, and increased access to and quality of medical care. Policies guiding the utilization of wild birds and animals as a source of food need to be reviewed to reduce prospects for zoonotic transmission. Continued surveillance is warranted to identify any potential changes in circulating RV genotype and atypical strain that are not targeted by the current vaccine. The molecular basis underlying the strong interaction of rotavirus with the host gastrointestinal tract relies on the interplay between a variety of cellular and viral factors, some of which may be potentially targeted by future vaccines or therapies.

## Figures and Tables

**Table 1:** The number of genotypes ascribed to each gene segment (updated) and the biological functions of their encoded proteins [25,26].

Genome Segment	Size (bp)	Number of Genotype	Genotype Denotation	Protein Product	Type of Protein/Location in Virion	Function
1	3302	22	R	VP1	Structural, inner capsid	-RdRp -ss-RNA binding
2	2687	20	C	VP2	Structural, core	-Houses RNA genome
3	2592	20	M	VP3	Structrural, inner capsid	-guanyltransferase -methyltransferase -ss RNA binding
4	2362	51	P	VP4	Structural, outer capsid	-receptor binding protein -infectivity enhancement through trysin cleavage
5	1356	26	I	VP6	Structural, middle capsid	-Serological grouping and subgrouping antigen
6	1062	36	G	VP7	Structural, outer capsid	-Neutralization antigen -Bases of binary classification
7	1581	31	A	NSP1	Non-structural	-host interferon antagonist -anti-apoptosis
8	1059	22	N	NSP2	Non-structural	-helicase -NTPase -NDPK -RBP
9	1074	22	T	NSP3	Non-structural	-competition with host PABP for elf-4G1 binding -Translation enhancer
10	751	27	E	NSP4	Non-structural	-enterotoxin -Transmembrane gp
11	666	22	H	NSP5	Non-structural	-phosphoprotein
				NSP6	Non-structural	-ssRNA and dsRNA binding

Note: RdRp = RNA dependent RNA polymerase; PABP = poly (A) binding protein; RBP = RNA binding protein; NDPK = Nucleoside diphosphate kinases.

**Table 2:** Characteristics of rotavirus vaccines approved for use.

Name	Licensing	Date of WHO Prequalification	Vaccine Antigens	Formulation	Storage Conditions	No. of Doses	Schedule	References
Rotarix (RV1; GSK)	Globally	March, 2009	Live-attenuated, human wild-type G1P[8] strain [R1X4414]	Liquid	2–8 °C for 36 months	2	2 months and 4 months	[60,171]
RotaTeq (RV5; Merck)	Globally	October, 2008	Live-attenuated, human-bovine rotavirus reassortant G1, G2, G3, G4 and P[8]	Liquid	2–8 °C for 36 months	3	2 months, 4 months and 6 months	[170]
Rotavac (Bharat)	Globally	January, 2018	Live-attenuated wild-type reassortant G9P[11] strain [116E]	Liquid frozen	2–8 °C for 7 months, −20 °C (long-term)	3	6 weeks, 10 weeks and 14 weeks	[78]
Rotasiil (Serum institute)	Globally	September, 2018	Live-attenuated human-bovine rotavirus reassortant G1, G2, G3, G4, and G9	Lyophilized, Thermostable lyophilized & Liquid	<40 °C for 18 months <25 °C for 30 months 14 weeks	3	6 weeks, 10 weeks and 14 weeks	[4,22]
Rotavin-M1 (POLYVAC)	Nationally	Not yet	Live-attenuated human rotavirus strain G1P[8]	Liquid frozen	2–8 °C for 2 months −20 °C for 24 months	2	Minimum at 6 weeks, for 4 weeks apart	[4,22]
Lanzhou lamb (Lanzhou institute)	Nationally	Not yet	Live-attenuated lamb G10P[15] rotavirus strain	Liquid	2–8 °C for 12 months	4	I dose annually for children aged 2–36 months	[4,22,60]

**Table 3:** Efficacy data for globally licensed rotavirus vaccines.

Name	Region	Efficacy (95% CI)	Reference
Rotarix	Europe	96% (90–99%)	[182]
	Latin America	85% (72–92%)	[183]
	Africa	62% (44–73%)	[184]
Rotateq	Europe	98% (88–100%)	[185]
	Africa	64% (40–79%)	[186]
	Asia	51% (13–73%)	[187]
Rotavac	Asia	54% (37–70%)	[188]
Rotasiil	Africa	67% (50–78%)	[189]
Rotasiil	Asia	36% (12–54%)	[190]

**Table 4:** Potential anti-rotavirus drugs.

Name	Mechanism	Reference
Gemcitabine	Pyrimidine nucleotide inhibitor	[242]
2′-*C*-methyl nucleosides	Viral polymerase inhibitor	[243]
Racecadotril	Intestinal encephalinase inhibitor	[244]
Nitazoxanide	Inhibitor of viroplasm formation	[245]
Resveratrol	Inhibitor of viral protein synthesis	[249]
Ziyuglycoside II	Inhibitor of TLR4/NF-κB pathway	[246]
Brequinar	Pyrimidine biosynthesis inhibitor	[248]
ML-60218	RNA polymerase III inhibitor	[252]
Genipin	Entry inhibitor	[253]

## Data Availability

Data used in the study are included within the text.

## References

[1] Troeger C., Khalil I.A., Rao P.C., Cao S., Blacker B.F., Ahmed T., Armah G., Bines J.E., Brewer T.G., Colombara D.V. (2018). Rotavirus Vaccination and the Global Burden of Rotavirus Diarrhea among Children Younger Than 5 Years. JAMA Pediatr..

[2] Tate J.E., Burton A.H., Boschi-Pinto C., Parashar U.D., World Health Organization–Coordinated Global Rotavirus Surveillance Network (2016). Global, Regional, and National Estimates of Rotavirus Mortality in Children <5 Years of Age, 2000–2013. Clin. Infect. Dis..

[3] Moraga P., GBD 2016 Causes of Death Collaborators (2017). Global, regional, and national age-sex specific mortality for 264 causes of death, 1980–2016: A systematic analysis for the Global Burden of Disease Study 2016. Lancet.

[4] World Health Organization Summary of Key Characteristics of Currently WHO-Pre-Qualified Rotavirus Vaccines, Version 1.4 Dated 26 February 2019. https://www.who.int/publications/i/item/WHO-IVB-2021.03.

[5] Kim A.H., Hogarty M.P., Harris V.C., Baldridge M.T. (2021). The Complex Interactions between Rotavirus and the Gut Microbiota. Front. Cell Infect. Microbiol..

[6] Lee B. (2021). Update on rotavirus vaccine underperformance in low- to middle-income countries and next-generation vaccines. Hum. Vaccines Immunother..

[7] Saha D., Ota M.O.C., Pereira P., Buchy P., Badur S. (2021). Rotavirus vaccines performance: Dynamic interdependence of host, pathogen and environment. Expert Rev. Vaccines.

[8] Adams W.R., Kraft L.M. (1963). Epizootic diarrhea of infant mice: Indentification of the etiologic agent. Science.

[9] Bishop R.F., Davidson G.P., Holmes I.H., Ruck B.J. (1973). Virus particles in epithelial cells of duodenal mucosa from children with viral gastroenteritis. Lancet.

[10] Bishop R.F., Davidson G.P., Holmes I.H., Ruck B.J. (1974). Detection of a new virus by electron microscopy of faecal extracts from children with acute gastroenteritis. Lancet.

[11] Flewett T.H., Woode G.N. (1978). The rotaviruses. Arch. Virol..

[12] Estes M.K., Fields B.N., Knipe D.M., Howley P.M., Chanock R.M., Monath T.P., Melnick J.L. (1996). Rotaviruses and their replication. Fields Virology.

[13] National Center for Immunization and Respiratory Diseases/CDC Rotavirus Vaccination. Last Reviewed: 25 July 2018. https://www.cdc.gov/vaccines/vpd/rotavirus/index.html.

[14] World Health Organization Causes of Child Mortality (WHO 2021). https://www.who.int/gho/child_health/mortality/causes/en/.

[15] Long C.P., McDonald S.M. (2017). Rotavirus genome replication: Some assembly required. PLoS Pathog..

[16] Estes M.K., Kapikian A., Knipe D., Griffin D., Lamb R., Martin M., Roizman B., Straus S. (2007). Rotaviruses. Fields Virology.

[17] Gómez-Rial J., Rivero-Calle I., Salas A., Martinón-Torres F. (2020). Rotavirus and autoimmunity. J. Inf..

[18] Surendran S. (2008). Review article: Rotavirus infection: Molecular changes and Pathophysiology. EXCLI J..

[19] Crawford S.E., Ramani S., Tate J.E., Parashar U.D., Svensson L., Hagbom M., Franco M.A., Greenberg H.B., O’Ryan M., Kang G. (2017). Rotavirus infection. Nat. Rev. Dis. Primers.

[20] Matthijnssens J., Otto P.H., Ciarlet M., Desselberger U., Van Ranst M., Johne R. (2012). VP6-sequence-based cutoff values as a criterion for rotavirus species demarcation. Arch. Virol..

[21] Steger C.L., Boudreaux C.E., LaConte L.E., Pease J.B., McDonald S.M. (2019). Group a Rotavirus VP1 Polymerase and VP2 Core Shell Proteins: Intergenotypic Sequence Variation and In Vitro Functional Compatibility. J. Virol..

[22] World Health Organization (WHO) (2021). Rotavirus Vaccines: WHO Position Paper—July 2021. Wkly. Epidemiol. Rec..

[23] Matthijnssens J., Ciarlet M., McDonald S.M., Attoui H., Bányai K., Brister J.R. (2011). Uniformity of rotavirus strain nomenclature proposed by the Rotavirus Classification Working Group (RCWG). Arch. Virol..

[24] Cunliffe N.A., Bresee J.S., Gentsch J.R., Glass R.I., Hart C.A. (2002). The expanding diversity of rotaviruses. Lancet.

[25] Matthijnssens J., Ciarlet M., Heiman E., Arijs I., Delbeke T., McDonald S.M., Palombo A.E., Iturriza-Gómara M., Maes P., Patton J.T. (2008). Full genome-based classification of rotaviruses reveals common origin between human Wa-like and porcine rotavirus strains and human DS-1-like and bovine rotavirus strains. J. Virol..

[26] RCWG (2019). Newly Assigned Genotypes: List of Accepted Genotypes. https://rega.kuleuven.be/cev/viralmetagenomics/virus-classification/rcwg.

[27] World Health Organization (WHO) Vaccine-Preventable Diseases Surveillance Standards. Rotavirus. Last Updated: 5 September 2018. https://www.who.int/publications/i/item/surveillance-standards-for-vaccine-preventable-diseases-2nd-edition.

[28] Julian T.R. (2016). Environmental transmission of diarrheal pathogens in low and middle income countries. Environ. Sci. Process. Impacts.

[29] Centers for Disease Control and Prevention (2015). Sustained Decrease in Laboratory Detection of Rotavirus after Implementation of Routine Vaccination—United States 2004–2014. CDC. https://www.cdc.gov/mmwr/preview/mmwrhtml/mm6413a1.htm.

[30] Burke R.M., Tate J.E., Barin N., Bock C., Bowen M.D., Chang D., Gautam R., Han G., Holguin J., Huynh T. (2018). Three rotavirus outbreaks in the postvaccine era—California, 2017. MMWR Morb. Mortal. Wkly. Rep..

[31] Wilde J., Van R., Pickering L., Eiden J., Yolken R. (1992). Detection of rotaviruses in the day care environment by reverse transcriptase polymerase chain reaction. J. Infect. Dis..

[32] Meštrović T. Rotavirus Transmission. News Medical.Net. Last Updated: 2 March 2021. https://www.newsmedical.net/health/Rotavirus-Transmission.aspx.

[33] Dennehy P.H. (2000). Transmission of rotavirus and other enteric pathogens in the home. Pediatr. Infect. Dis. J..

[34] Rogers M., Weinstock D.M., Eagan J., Kiehn T., Armstrong D., Sepkowitz K.A. (2000). Rotavirus outbreak on a pediatric oncology floor: Possible association with toys. Amer. J. Inf. Control..

[35] Cortese M.M., Haber P., Centers for Disease Prevention and Control (2005). Epidemiology and Prevention of Vaccine-Preventable Diseases.

[36] Abad F.X., Pinto R.M., Bosch A. (1994). Survival of enteric viruses on environmental fomites. Appl. Environ. Microbiol..

[37] Gallimore C.I., Pipkin C., Shrimpton H., Green A.D., Pickford Y., McCartney C. (2005). Detection of multiple enteric virus strains within a foodborne outbreak of gastroenteritis: An indication of the source of contamination. Epidemiol. Infect..

[38] Quiroz-Santiago C., Vázquez-Salinas C., Natividad-Bonifacio I., Barrón-Romero B.L., Quiñones-Ramírez E.I. (2014). Rotavirus G2P4 detection in fresh vegetables and oysters in Mexico City. J. Food Prot..

[39] van Zyl W.B., Page N.A., Grabow W.O.K., Steele A.D., Taylor M.B. (2006). Molecular Epidemiology of Group A Rotaviruses in Water Sources and Selected Raw Vegetables in Southern Africa. Appl. Environ. Microbiol..

[40] NCID, National Institute of Infectious Diseases (2000). An Outbreak of Group A Rotavirus Infections among Adults from Eating Meals Prepared at a Restaurant, April 2000—Shimane.

[41] Le Guyader F.S., Le Saux J.C., Ambert-Balay K., Krol J., Serais O., Parnaudeau S., Giraudon H., Delmas G., Pommepuy M., Pothier P. (2008). Aichi virus, norovirus, astrovirus, enterovirus, and rotavirus involved in clinical cases from a French oyster-related gastroenteritis outbreak. J. Clin. Microbiol..

[42] Iritani N., Kaida A., Abe N., Kubo H., Sekiguchi J., Yamamoto S.P., Goto K., Tanaka T., Noda M. (2014). Detection and genetic characterization of human enteric viruses in oyster-associated gastroenteritis outbreaks between 2001 and 2012 in Osaka City, Japan. J. Med. Virol..

[43] Centers for Disease Control and Prevention (CDC) (2000). Foodborne outbreak of Group A rotavirus gastroenteritis among college students—District of Columbia, March–April 2000. MMWR Morb. Mortal. Wkly. Rep..

[44] Mizukoshi F., Kuroda M., Tsukagoshi H., Sekizuka T., Funatogawa K., Morita Y., Noda M., Katayama K., Kimura H. (2014). A food-borne outbreak of gastroenteritis due to genotype G1P[8] rotavirus among adolescents in Japan. Microbiol. Immunol..

[45] Fleet G.H., Heiskanen P., Reid I., Buckle K.A. (2000). Foodborne viral illness—Status in Australia. Int. J. Food Microbiol..

[46] Mayr C., Strohe G., Contzen M. (2009). Detection of rotavirus in food associated with a gastroenteritis outbreak in a mother and child sanatorium. Int. J. Food Microbiol..

[47] Ansari S.A., Sattar S.A., Springthorpe V.S., Wells G.A., Tostowaryk W. (1988). Rotavirus survival on human hands and transfer of infectious virus to animate and nonporous inanimate surfaces. J. Clin. Microbiol..

[48] Gastañaduy P.A., Hall A.J., Parashar U.D. (2013). Rotavirus. Foodborne Infect. Intox..

[49] U.S. EPA (2011). Exposure Factors Handbook 2011 Edition (Final).

[50] Omatola C.A., Olusola B.A., Odaibo G.N. (2016). Rotavirus infection among under five children presenting with gastroenteritis in Ibadan, Nigeria. Arch. Bas. Appl. Med..

[51] CDC (Centers for Disease Control and Prevention) (1998–2009) Foodborne Disease Outbreak Surveillance System (FDOSS), 1998–2009 Atlanta, Georgia. https://www.cdc.gov/fdoss/index.html.

[52] Tan S.W., Yap K.L., Lee H.L. (1997). Mechanical Transport of Rotavirus by the Legs and Wings of *Musca domestica* (Diptera: Muscidae). J. Med. Entomol..

[53] Issa R. (2019). Musca domestica acts as transport vector hosts. Bull. Natl. Res. Cent..

[54] Collinet-Adler S., Babji S., Francis M., Kattula D., Premkumar P.S., Sarkar R., Mohan V.R., Ward H., Kang G., Balraj V. (2015). Environmental Factors Associated with High Fly Densities and Diarrhea in Vellore, India. Appl. Environ. Microbiol..

[55] Dóró R., Farkas S.L., Martella V., Bányai K. (2015). Zoonotic transmission of rotavirus: Surveillance and control. Expert Rev. Anti. Infect. Ther..

[56] Rojas M., Dias H.G., Gonçalves J.L.S., Manchego A., Rosadio R., Pezo D., Santos N. (2019). Genetic diversity and zoonotic potential of rotavirus A strains in the southern Andean highlands, Peru. Transbound. Emerg. Dis..

[57] Malik Y.S., Bhat S., Dar P.S., Sircar S., Dhama K., Singh R.K. (2020). Evolving Rotaviruses, Interspecies Transmission and Zoonoses. Open Virol. J..

[58] Omatola C.A., Olaniran A.O. (2022). Epidemiological significance of the occurrence and persistence of rotaviruses in water and sewage: A critical review and proposal for routine microbiological monitoring. Environ. Sci. Processes Impacts.

[59] Harris A.R., Davis J., Boehm A.B. (2013). Mechanisms of post-supply contamination of drinking water in Bagamoyo, Tanzania. J. Water Health.

[60] Yen C., Cortese M.M. (2018). Rotaviruses. Principles and Practice of Pediatric Infectious Diseases.

[61] Bishop R.F., Masendycz P.J., Bugg H.C. (2001). Epidemiological patterns of rotaviruses causing severe gastroenteritis in young children throughout Australia from 1993 to 1996. J. Clin. Microbiol..

[62] Levy K., Hubbard A.E., Elsenberg J.N.S. (2009). Seasonality of rotavirus disease in the tropics: A systematic review and meta-analysis. Int. J. Epidemiol..

[63] Fragoso M., Kumar A., Murray D.L. (1986). Rotavirus in nasopharyngeal secretions of children with upper respiratory tract infections. Diagn. Microbiol. Infect. Dis..

[64] Zhen B.J., Chang R.X., Ma G.Z., Xie J.M., Liu Q., Liang X.R., Ng M.H. (1991). Rotavirus infection of the oropharynx and respiratory tract in young children. J. Med. Virol..

[65] Goldwater P., Chrystie I., Banatvala J. (1979). Rotaviruses and the respiratory tract. Br. Med. J..

[66] Grimwood K., Lambert S.B., Milne R.J. (2010). Rotavirus infections and vaccines: Burden of illness and potential impact of vaccination. Paediatr. Drugs.

[67] Ginn O., Rocha-Melogno L., Bivins A., Lowry S., Cardelino M., Nichols D., Tripathi S., Soria F., Andrade M., Bergin M. (2021). Detection and quantification of enteric pathogens in aerosols near open wastewater canals in cities with poor sanitation. MedRxiv.

[68] Okaali D.A., Hofstra N. (2018). Present and Future Human Emissions of Rotavirus and to Uganda’s Surface Waters. J. Environ. Quality..

[69] Bosch A. (1998). Human enteric viruses in the water environment: A minireview. Int. Microbiol..

[70] Estes M.K., Greenberg H.B., Knipe D.M., Howley P.M. (2013). Rotaviruses. Fields Virology.

[71] Sadiq A., Bostan N., Yinda K.C., Naseem S., Sattar S. (2018). Rotavirus: Genetics, pathogenesis and vaccine advances. Rev. Med. Virol..

[72] Saxena K., Blutt S.E., Ettayebi K., Zeng X.L., Broughman J.R., Crawford S.E., Karandikar U.C., Sastri N.P., Conner M.E., Opekun A.R. (2015). Human intestinal enteroids: A new model to study human rotavirus infection, host restriction, and pathophysiology. J. Virol..

[73] Tan M., Jiang X. (2014). Histo-blood group antigens: A common niche for norovirus and rotavirus. Expert Rev. Mol. Med..

[74] Arias C.F., Silva-Ayala D., López S. (2015). Rotavirus entry: A deep journey into the cell with several exits. J. Virol..

[75] Ramani S., Hu L., Venkataram Prasad B.V., Estes M.K. (2016). Diversity in rotavirus-host glycan interactions: A “sweet” spectrum. Cell. Mol. Gastroenterol. Hepatol..

[76] Sharma S., Hagbom M., Svensson L., Nordgren J. (2020). The Impact of Human Genetic Polymorphisms on Rotavirus Susceptibility, Epidemiology, and Vaccine Take. Viruses.

[77] Heggelund J.E., Varrot A., Imberty A., Krengel U. (2017). Histo-blood group antigens as mediators of infections. Curr. Opin. Struct. Biol..

[78] Cárcamo-Calvo R., Muñoz C., Buesa J., Rodríguez-Díaz J., Gozalbo-Rovira R. (2021). The Rotavirus Vaccine Landscape, an Update. Pathogens.

[79] Imbert-Marcille B.M., Barbé L., Dupé M., Le Moullac-Vaidye B., Besse B., Peltier C., Ruvoën-Clouet N., Le Pendu J. (2013). A FUT2 gene common polymorphism determines resistance to Rotavirus A of the P[8] genotype. J. Infect. Dis..

[80] Huang P., Xia M., Tan M., Zhong W., Wei C., Wang L., Morrow A., Jiang X. (2012). Spike protein VP8* of human rotavirus recognizes histo-blood group antigens in a type-specific manner. J. Virol..

[81] Liu Y.Y., Huang P., Tan M., Biesiada J., Meller J., Castello A.A., Jiang B., Jiang X. (2012). Rotavirus VP8*: Phylogeny, host range, and interaction with histo-blood group antigens. J. Virol..

[82] Liu Y., Jiang X., Huang P., Jiang B., Tan M., Morrow A.L. (2013). Poly-LacNAc as an Age-Specific Ligand for Rotavirus P[11] in Neonates and Infants. PLoS ONE.

[83] Khambhampati A., Payne D.C., Constantini V., Lopman B.A. (2016). Host genetic susceptibility to enteric viruses: A systematic review and meta-analysis. Clin. Infect. Dis..

[84] Guo L.-A., Zhang M., Hou Y.Z., Hu H., Fang L., Tan M., Huang Q., Li H., Sun L.-M., Jiang X. (2020). Epidemiology and HBGA-susceptibility investigation of a G9P[8] rotavirus outbreak in a school in Lechang, China. Arch. Virol..

[85] Cantelli C.P., Velloso A.J., Assis R.M.S., de-Barros J.J., Mello F.C.d.A., Cunha D.C.d., Brasil P., Nordgren J., Svensson L., Miagostovich M.P. (2020). Rotavirus A shedding and HBGA host genetic susceptibility in a birth community-cohort, Rio de Janeiro, Brazil, 2014–2018. Sci. Rep..

[86] Li B., Ding S., Feng N., Mooney N., Ooi Y.S., Ren L., Diep J., Kelly M.R., Yasukawa L.L., Patton J.T. (2017). Drebrin restricts rotavirus entry by inhibiting dynamin-mediated endocytosis. Proc. Natl. Acad. Sci. USA.

[87] Herrmann T., Torres R., Salgado E.N., Berciu C., Stoddard D., Nicastro D., Jenni S., Harrison S.C. (2021). Functional refolding of the penetration protein on a non-enveloped virus. Nature.

[88] Kapikian A.Z., Chanock R.M., Fields B.N., Knipe D.M., Howley P.M., Chanock R.M., Monath T.P., Melnick J.L. (1996). Rotaviruses. Fields Virology.

[89] O’Ryan M.G. Clinical Manifestations and Diagnosis of Rotavirus Infection: An Update. Wolters Kluwers. https://www.uptodate.com/contents/clinical-manifestations-and-diagnosis-of-rotavirus-infection#H3096970958.

[90] Hyams J.S., Krause P.J., Gleason P.A. (1981). Lactose malabsorption following rotavirus infection in young children. J. Pediatr..

[91] Seo N.-N., Carl Q.-Y., Utama B., Crawford S.E., Kim K.J., Höök M., Estes M.K. (2008). Integrins α1β1 and α2β1 are receptors for the rotavirus enterotoxin. Proc. Natl. Acad. Sci. USA.

[92] Hyser J.M., Collinson-Pautz M.R., Utama B., Estes M.K. (2010). Rotavirus disrupts calcium homeostasis by NSP4 viroporin activity. MBio.

[93] Farnworth E.R. (2008). The evidence to support health claims for probiotics. J. Nutr..

[94] Arya S.C. (1984). Rotaviral Infection and Intestinal Lactase Level. J. Infect. Dis..

[95] Bialowas S., Hagbom M., Nordgren J., Karlsson T., Sharma S., Magnusson K.E., Svensson L. (2016). Rotavirus and serotonin cross-talk in diarrhoea. PLoS ONE.

[96] Lundgren O., Peregrin A.T., Persson K., Kordasti S., Uhnoo I., Svensson L. (2000). Role of the enteric nervous system in the fluid and electrolyte secretion of rotavirus diarrhea. Science.

[97] Bass E.S., Pappano D.A., Humiston S.G. (2007). Rotavirus. Pediatr. Rev..

[98] Marie H., Claudia I., David E., Thommie K., Jesus R., Javier B., John A.T. (2011). Rotavirus stimulates release of serotonin from human enterochromaffin cells and activates brain cells involved in nausea and vomiting. PLoS Pathog..

[99] Ray P., Fenaux M., Sharma S., Malik J., Subodh S., Bhatnagar S., Greenberg H., Glass R.I. (2006). Quantitative evaluation of rotaviral antigenemia in children with acute rotaviral diarrhea. J. Infect Dis..

[100] Gómez-Rial J., Sánchez-Batán S., Rivero-Calle I., Pardo-Seco J., Martinón-Martínez J.M., Salas A., Martinón-Torres F. (2019). Rotavirus infection beyond the gut. Infect. Drug Resist..

[101] Rivero-Calle I., Gómez-Rial J., Martinón-Torres F. (2016). Systemic features of rotavirus infection. J. Inf..

[102] Hemming M., Huhti L., Räsänen S., Salminen M., Vesikari T. (2014). Rotavirus antigenemia in children is associated with more severe clinical manifestations of acute gastroenteritis. Pediatr. Infect. Dis. J..

[103] Sugata K., Taniguchi K., Yui A., Miyake F., Suga S., Asano Y., Ohashi M., Suzuki K., Nishimura N., Osaki T. (2008). Analysis of rotavirus antigenemia and extraintestinal manifestations in children with rotavirus gastroenteritis. Pediatrics.

[104] (2010). Canada Communicable Disease Report (CCDR). Literature Review on Rotavirus: Disease and Vaccine Characteristics. An Advisory Committee Statement (ACS) by National Advisory Committee on Immunization (NACI).

[105] Payne D.C., Parashar U.D. (2017). Rotavirus. VPD Surveillance Manual Rotavirus: Chapter 13.1. https://www.cdc.gov/vaccines/pubs/surv-manual/chpt13-rotavirus.pdf.

[106] Kumar A., Vlasova A.N., Deblais L., Huang H.C., Wijeratne A., Kandasamy S., Fischer D.D., Langel S.N., Paim F.C., Alhamo M.A. (2018). Impact of nutrition and rotavirus infection on the infant gut microbiota in a humanized pig model. BMC Gastroenterol..

[107] Gonzalez-Ochoa G., Flores-Mendoza L.K., Icedo-Garcia1 R., Gomez-Flores R., Tamez-Guerra P. (2017). Modulation of rotavirus severe gastroenteritis by the combination of probiotics and prebiotics. Arch. Microbiol..

[108] Athiyyah A.F., Utsumi T., Wahyuni R.M., Dinana Z., Yamani L.N., Sudarmo S.M., Ranuh R.G., Darma A., Juniastuti J., Raharjo D. (2019). Molecular Epidemiology and Clinical Features of Rotavirus Infection among Pediatric Patients in East Java, Indonesia during 2015–2018: Dynamic Changes in Rotavirus Genotypes from Equine-Like G3 to Typical Human G1/G3. Front. Microbiol..

[109] Senecal M., Brisson M., Lebel M.H., Yaremko J., Wong R., Gallant L.A. (2006). Severity, healthcare resource use and work loss related to rotavirus gastroenteritis: A prospective study in community practice. Canadian Public Health Association. Vancouver.

[110] Leung A.K., Kellner J.D., Davies H.D. (2005). Rotavirus gastroenteritis. Adv. Ther..

[111] Center for Disease Control and Prevention/National Center for Immunization and Respiratory Diseases (CDC/NCIRD) Division of Viral Diseases. Updated: 5 November 2019. https://www.cdc.gov/.

[112] Glass R.I., Parashar U.D., Bresee J.S., Turcios R., Fischer T.K., Widdowson M.A., Jiang B. (2006). Rotavirus vaccines: Current prospects and future challenges. Lancet.

[113] Sen A., Greenberg H.B. (2016). Innate Immune Responses to Rotavirus Infection.

[114] Reikine S., Nguyen J.B., Modis Y. (2014). Pattern recognition and signaling mechanisms of RIG-I and MDA5. Front. Immunol..

[115] Arnold M.M., Patton J.T. (2011). Diversity of interferon antagonist activities mediated by NSP1 proteins of different rotavirus strains. J. Virol..

[116] Villena J., Vizoso-Pinto M.G., Kitazawa H. (2016). Intestinal Innate Antiviral Immunity and Immunobiotics: Beneficial Effects against Rotavirus Infection. Front. Immunol..

[117] Holloway G., Coulson B.S. (2013). Innate cellular responses to rotavirus infection. J. Gen. Virol..

[118] Uchiyama R., Chassaing B., Zhang B., Gewirtz A.T. (2015). MyD88-mediated TLR signaling protects against acute rotavirus infection while inflammasome cytokines direct Ab response. Innate Immun..

[119] Sánchez-Tacuba L., Rojas M., Arias C.F., López S. (2015). Rotavirus Controls Activation of the 2′-5′-Oligoadenylate Synthetase/RNase L Pathway Using at least Two Distinct Mechanisms. J. Virol..

[120] Song Y., Feng N., Sanchez-Tacuba L., Yasukawa L.L., Ren L., Silverman R.H., Ding S., Greenberg H.B. (2020). Reverse Genetics Reveals a Role of Rotavirus VP3 Phosphodiesterase Activity in Inhibiting RNase L Signaling and Contributing to Intestinal Viral Replication In Vivo. J. Virol..

[121] Zhu S., Ding S., Wang P., Wei Z., Pan W., Palm N.W., Yang Y., Yu H., Li H.B., Wang G. (2017). Nlrp9b inflammasome restricts rotavirus infection in intestinal epithelial cells. Nature.

[122] Desselberger U., Huppertz H.I. (2011). Immune responses to rotavirus infection and vaccination and associated correlates of protection. J. Infect. Dis..

[123] Patel M., Glass R.I., Jiang B., Santosham M., Lopman B., Parashar U. (2013). A systematic review of anti-rotavirus serum IgA antibody titer as a potential correlate of rotavirus vaccine efficacy. J. Infect. Dis..

[124] Franco M.A., Angel J., Greenberg H.B. (2006). Immunity and correlates of protection for rotavirus vaccines. Vaccine.

[125] Green K.Y., Taniguchi K., Mackow E.R., Kapikian A.Z. (1990). Homotypic and heterotypic epitope-specific antibody responses in adult and infant rotavirus vaccinees: Implications for vaccine development. J. Infect. Dis..

[126] Velazquez F.R., Matson D.O., Guerrero M.L., Shults J., Calva J.J., Morro A.L. (2000). Natural Immunity to Rotavirus Infection in Children. J. Infect. Dis..

[127] Matson D.O., O’Ryan M.L., Herrera I., Pickering L.K., Estes M.K. (1993). Fecal antibody responses to symptomatic asymptomatic rotavirus infections. J. Infect. Dis..

[128] Sinha A., Kanungo S., Kim D.R., Manna B., Song M., Park J.Y., Haldar B., Sharma P., Mallick A.H., Kim S.A. (2018). Antibody secreting B cells and plasma antibody response to rotavirus vaccination in infants from Kolkata India. Heliyon.

[129] Caddy S.L., Vaysburd M., Wing M., Foss S., Andersen J.T., O’Connell K. (2020). Intracellular neutralisation of rotavirus by VP6-specific IgG. PLoS Pathog.

[130] Malm M., Hyöty H., Knip M., Vesikari T., Blazevic V. (2019). Development of T cell immunity to norovirus and rotavirus in children under five years of age. Sci. Rep..

[131] Smiley K.L., McNeal M.M., Basu M., Choi A.H., Clements J.D., Ward R.L. (2007). Association of gamma interferon and interleukin-17 production in intestinal CD4^+^ T cells with protection against rotavirus shedding in mice intranasally immunized with VP6 and the adjuvant LT(R192G). J. Virol..

[132] Godefroy E., Alameddine J., Montassier E., Mathé J., Desfrançois-Noël J., Marec N., Bossard C., Jarry A., Bridonneau C., Le Roy A. (2018). Expression of CCR6 and CXCR6 by Gut-Derived CD4^+^/CD8α^+^ T-Regulatory Cells, Which Are Decreased in Blood Samples from Patients with Inflammatory Bowel Diseases. Gastroenterology.

[133] Makela M., Marttila J., Simell O., Ilonen J. (2004). Rotavirus-specific T-cell responses in young prospectively followed-up children. Clin. Exp. Immunol..

[134] Franco M.A., Greenberg H.B. (1997). Immunity to rotavirus in T cell deficient mice. Virology.

[135] Garcia-Sastre A., Biron C.A. (2006). Type 1 interferons and the virus-host relationship: A lesson in detente. Science.

[136] Simon A.K., Hollander G.A., McMichael A. (2015). Evolution of the immune system in humans from infancy to old age. Proc. Biol. Sci..

[137] Xu J., Dennehy P., Keyserling H., Westerman L.E., Wang Y., Holman R.C., Gentsch J.R., Glass R.I., Jiang B. (2005). Serum Antibody Responses in Children with Rotavirus Diarrhea Can Serve as Proxy for Protection. Clin. Diagn. Lab. Immunol..

[138] Villamizar-Gallardo R.A., Osma J.F., Ortíz O.O. (2017). New technique for direct fluoroimmunomagnetic detection of rotavirus in water samples. J. Water Health.

[139] World Health Organization (WHO) (2009). Rotavirus vaccines: An update. Wkly. Epidemiol. Rec..

[140] El-Ageery M.S., Ali R., El-Khier N.T.A., Rakha S.A., Zeid M.S. (2020). Comparison of enzyme immunoassay, latex agglutination and polyacrylamide gel electrophoresis for diagnosis of rotavirus in children. Egypt. J. Basic Appl. Sci..

[141] Carossino M., Barrandeguy M.E., Erol E., Li Y., Balasuriya U. (2019). Development and evaluation of a one-step multiplex real-time TaqMan^®^ RT-qPCR assay for the detection and genotyping of equine G3 and G14 rotaviruses in fecal samples. Virol. J..

[142] Tate J.E., Burton A.H., Boschi-Pinto C., Steele A.D., Duque J., Parashar U.D. (2012). WHO-coordinated Global Rotavirus Surveillance Network. 2008 estimate of worldwide rotavirus-associated mortality in children younger than 5 years before the introduction of universal rotavirus vaccination programmes: A systematic review and meta-analysis. Lancet Infect. Dis..

[143] Badura S., Öztürka S., Pereira P., AbdelGhanyc M., Khalafd M., Lagoubie Y., Hanifg O.K., Saha D. (2019). Systematic review of the rotavirus infection burden in the WHO-EMRO region. Hum. Vaccines Immunother..

[144] Parashar U.D., Burton A., Lanata C., Boschi-Pinto C., Shibuya K., Steele D., Birmingham M., Glass R.I. (2009). Global mortality associated with rotavirus disease among children in 2004. J. Infect. Dis..

[145] Diez-Domingo J., Garces-Sanchez M., Gimenez-Sanchez F., Colomina-Rodriguez J., Martinon-Torres F. (2019). What have we learnt about rotavirus in Spain in the last 10 years?. An. Pediatr..

[146] (2017). Rota Council. Global Introduction Status. http://rotacouncil.org/vaccine-introduction/globalintroduction-status/.

[147] Ardura-Garcia C., Kreis C., Rakic M., Jaboyedoff M., Mallet M.C., Low N., Kuehni C.E. (2021). Rotavirus disease and health care utilisation among children under 5 years of age in highly developed countries: A systematic review and *meta*-analysis. Vaccine.

[148] Anderson E.J., Weber S.G. (2004). Rotavirus infection in adults. Lancet Infect. Dis..

[149] Linhares A.C., Pinheiro F.P., Freitas R.B., Gabbay Y.B., Shirley J.A., Beards G.M. (1981). An outbreak of rotavirus diarrhea among a non-immune, isolated South American Indian community. Am. J. Epidemiol..

[150] Bucardo F., Karlsson B., Nordgren J., Paniagua M., González A., Amador J.J., Espinoza F., Svensson L. (2007). Mutated G4P[8] rotavirus associated with a nationwide outbreak of gastroenteritis in Nicaragua in 2005. J. Clin. Microbiol..

[151] WHO Africa. WHO Supports Botswana to Respond to an Outbreak of Diarrhoea in Children below Five Years of Age. 29 October 2018. https://www.afro.who.int/news/who-supports-botswana-respond-outbreak-diarrhoea-children-below-five-years-age.

[152] Lahon A., Maniya N.H., Tambe G.U., Chinchole P.R., Purwar S., Jacob G., Chitambar S.D. (2013). Group B rotavirus infection in patients with acute gastroenteritis from India: 1994–1995 and 2004–2010. Epidemiol. Infect..

[153] Omatola C.A., Ogunsakin R.E., Olaniran A.O. (2021). Prevalence, Pattern and Genetic Diversity of Rotaviruses among Children under 5 Years of Age with Acute Gastroenteritis in South Africa: A Systematic Review and Meta-Analysis. Viruses.

[154] Li K., Lin X.D., Huang K.Y., Zhang B., Shi M., Guo W.P., Wang M.R., Wang W., Xing J.G., Li M.H. (2016). Identification of novel and diverse rotaviruses in rodents and insectivores, and evidence of cross-species transmission into humans. Virology.

[155] World Health Organization (WHO) (2011). Global Rotavirus Information and Surveillance Bulletin. Reporting Period: January through December 2010.

[156] Seheri L.M., Magagula N.B., Peenze I., Rakau K., Ndadza A., Mwenda J.M., Weldegebriel G., Steele A.D., Mphahlele M.J. (2018). Rotavirus strain diversity in Eastern and Southern African countries before and after vaccine introduction. Vaccine.

[157] Rakau K.G., Nyaga M.M., Gededzha M.P., Mwenda J.M., Mphahlele M.J., Seheri L.M., Steele A.D. (2021). Genetic characterization of G12P[6] and G12P[8] rotavirus strains collected in six African countries between 2010 and 2014. BMC Infect. Dis..

[158] Azemi M., Berisha M., Ismaili-Jaha V., Kolgeci S., Avdiu M., Jakupi X., Hoxha R., Hoxha-Kamberi T. (2013). Socio-demographic, clinical and laboratory features of rotavirus gastroenteritis in children treated in pediatric clinic. Mat. Soc. Med..

[159] Wobudeya E., Bachou H., Karamagi C.K., Kalyango J.N., Mutebi E., Wamani H. (2011). Breastfeeding and the risk of rotavirus diarrhea in hospitalized infants in Uganda: A matched case control study. BMC Pediatr..

[160] de Waure C., Sarnari L., Chiavarini M., Ianiro G., Monini M., Alunno A., Camilloni B. (2020). 10-Year Rotavirus Infection Surveillance: Epidemiological Trends in the Pediatric Population of Perugia Province. Int. J. Environ. Res. Pub. Health.

[161] Lestari F.B., Vongpunsawad S., Wanlapakorn N., Poovorawan Y. (2020). Rotavirus infection in children in Southeast Asia 2008–2018: Disease burden, genotype distribution, seasonality, and vaccination. J. Biomed. Sci..

[162] Patel M., Pedreira C., De Oliveira L.H., Umaña J., Tate J., Lopman B., Sanchez E., Reyes M., Mercado J., Gonzalez A. (2012). Duration of protection of pentavalent rotavirus vaccination in Nicaragua. Pediatrics.

[163] Center for Disease Prevention and Control/National Center for Immunization and Respiratory Diseases, Division of Viral Diseases (CDC/NCIRD) Rotavirus Transmission. Page Last Reviewed: 26 March 2021. https://www.cdc.gov/rotavirus/about/transmission.html.

[164] Pitzer V.E., Viboud C., Lopman B.A., Patel M.M., Parashar U.D., Grenfell B.T. (2011). Influence of birth rates and transmission rates on the global seasonality of rotavirus incidence. J. R. Soc. Interface.

[165] Pitzer V.E., Viboud C., Simonsen L., Steiner C., Panozzo C.A., Alonso W.J., Miller M.A., Glass R.I., Glasser J.W., Parashar U.D. (2009). Demographic variability, vaccination, and the spatiotemporal dynamics of rotavirus epidemics. Science.

[166] Johansen K., Hedlund K.-O., Zweygberg-Wirgart B., Bennet R. (2008). Complications attributable to rotavirus-induced diarrhoea in a Swedish paediatric population: Report from an 11-year surveillance. Scand. J. Infect. Dis..

[167] Stefkovicov M., Simurka P., Jurackov L., Hudeckov H., Mad’ar R. (2008). Nosocomial rotaviral gastroenteritis in paediatric departments. Cent. Eur. J. Public Health.

[168] Tran A.N., Husberg M., Bennet R., Brytting M., Carlsson P., Eriksson M., Storsaeter J., Österlin B., Johansen K. (2018). Impact on affected families and society of severe rotavirus infections in Swedish children assessed in a prospective cohort study. Infect. Dis..

[169] Burke R.M., Tate J.E., Kirkwood C.D., Steele A.D., Parashar U.D. (2019). Current and new rotavirus vaccines. Curr. Opin. Infect. Dis..

[170] Vetter V., Gardner R.C., Debrus S., Benninghoff B., Pereira P. (2021). Established and new rotavirus vaccines: A comprehensive review for healthcare professionals [published online ahead of print, 19 February 2021]. Hum. Vaccin. Immunother..

[171] Soares-Weiser K., Bergman H., Henschke N., Pitan F., Cunliffe N. (2019). Vaccines for preventing rotavirus diarrhoea: Vaccines in use. Cochrane Database of Systematic Reviews. Cochrane Database Syst. Rev..

[172] Sengupta P. (2009). Rotavirus: The challenges ahead. Indian J Community Med..

[173] Dang D.A., Nguyen V.T., Vu D.T., Nguyen T.H., Nguyen D.M., Yuhuan W., Baoming J., Nguyen D.H., Le T.L., Rotavin-M1 Vaccine Trial Group (2012). A dose-escalation safety and immunogenicity study of a new live attenuated human rotavirus vaccine (Rotavin-M1) in Vietnamese children. Vaccine.

[174] Skansberg A., Sauer M., Tan M., Santosham M., Jennings M.C. (2021). Product review of the rotavirus vaccines ROTASIIL, ROTAVAC, and Rotavin-M1. Hum. Vaccine. Immunother..

[175] Li J., Zhang Y., Yang Y., Liang Z., Tian Y., Liu B., Gao Z., Jia L., Chen L., Wang Q. (2019). Effectiveness of Lanzhou lamb rotavirus vaccine in preventing gastroenteritis among children younger than 5 years of age. Vaccine.

[176] Centers for Disease Control and Prevention, National Center for Emerging and Zoonotic Infectious Diseases (NCEZID), Division of Foodborne, Waterborne, and Environmental Diseases (DFWED) Rotavirus and Drinking Water from Private Wells. Page Last Reviewed: 1 July 2019. https://www.cdc.gov/healthywater/drinking/private/wells/disease/rotavirus.html.

[177] Pindyck T., Tate J.E., Parashar U.D. (2018). A decade of experience with rotavirus vaccination in the United States—vaccine uptake, effectiveness, and impact. Expert Rev. Vaccines.

[178] Mphahlele M.J., Groome M.J., Page N.A., Bhagwandin N., Mwenda J.M., Steele A.D. (2021). A decade of rotavirus vaccination in Africa–Saving lives and changing the face of diarrhoeal diseases: Report of the 12th African Rotavirus Symposium. Vaccine.

[179] Uprety T., Wang D., Li F. (2021). Recent advances in rotavirus reverse genetics and its utilization in basic research and vaccine development. Arch. Virol..

[180] Philip A.A., Patton J.T. (2020). Expression of Separate Heterologous Proteins from the Rotavirus NSP3 Genome Segment Using a Translational 2A Stop-Restart Element. J. Virol..

[181] Philip A.A., Patton J.T. (2021). Rotavirus as an expression platform of domains of the SARS-CoV-2 spike protein. Vaccines.

[182] Vesikari T., Karvonen A., Prymula R., Schuster V., Tejedor J.C., Cohen R., Meurice F., Han H.H., Damaso S., Bouckenooghe A. (2007). Efficacy of human rotavirus vaccine against rotavirus gastroenteritis during the first 2 years of life in European infants: Randomised, double-blind controlled study. Lancet.

[183] Ruiz-Palacios G.M., Pérez-Schael I., Velázquez F.R., Abate H., Breuer T., Clemens S.C., Cheuvart B., Espinoza F., Gillard P., Innis B.L. (2006). Safety and Efficacy of an Attenuated Vaccine against Severe Rotavirus Gastroenteritis. N. Engl. J. Med..

[184] Madhi S.A., Cunliffe N.A., Steele D., Witte D., Kirsten M., Louw C., Ngwira B., Victor J.C., Gillard P.H., Cheuvart B.B. (2010). Effect of human rotavirus vaccine on severe diarrhea in African infants. N. Engl. J. Med..

[185] Vesikari T., Matson D.O., Dennehy P., Van Damme P., Santosham M., Rodriguez Z., Dallas M.J., Heyse J.F., Goveia M.G., Black S.B. (2006). Safety and Efficacy of a Pentavalent Human–Bovine (WC3) Reassortant Rotavirus Vaccine. N. Engl. J. Med..

[186] Armah G.E., Sow S.O., Breiman R.F., Dallas M.J., Tapia M.D., Feikin D.R., Binka F.N., Steele A.D., Laserson K.F., Ansah N.A. (2010). Efficacy of pentavalent rotavirus vaccine against severe rotavirus gastroenteritis in infants in developing countries in sub-Saharan Africa: A randomised, double-blind, placebo-controlled trial. Lancet.

[187] Zaman K., Dang D.A., Victor J.C., Shin S., Yunus M., Dallas M.J., Podder G., Vu D.T., Le T.P., Luby S.P. (2010). Efficacy of pentavalent rotavirus vaccine against severe rotavirus gastroenteritis in infants in developing countries in Asia: A randomised, double-blind, placebo-controlled trial. Lancet.

[188] Bhandari N., Rongsen-Chandola T., Bavdekar A., John J., Antony K., Taneja S., Goyal N., Kawade A., Kang G., Rathore S.S. (2014). Efficacy of a monovalent human-bovine (116E) rotavirus vaccine in Indian children in the second year of life. Vaccine.

[189] Isanaka S., Guindo O., Langendorf C., Seck M.A., Plikaytis B.D., Sayinzoga-Makombe N., McNeal M.M., Meyer N., Adehossi E., Djibo A. (2017). Efficacy of a Low-Cost, Heat-Stable Oral Rotavirus Vaccine in Niger. N. Engl. J. Med..

[190] Kulkarni P.S., Desai S., Tewari T., Kawade A., Goyal N., Garg B.S., Kanungo S., Kamat V., Kang G., SII BRV-PV author group (2017) (2017). A randomized Phase III clinical trial to assess the efficacy of a bovine-human reassortant pentavalent rotavirus vaccine in Indian infants. Vaccine.

[191] Church J.A., Rukobo S., Govha M., Lee B., Carmolli M.P., Chasekwa B., Prendergast A.J. (2019). The Impact of Improved Water, Sanitation, and Hygiene on Oral Rotavirus Vaccine Immunogenicity in Zimbabwean Infants: Substudy of a Cluster-randomized Trial. Clin. Infect. Dis..

[192] Vlasova A.N., Takanashi S., Miyazaki A., Rajashekara G., Saif L.J. (2019). How the gut microbiome regulates host immune responses to viral vaccines. Curr. Opin. Virol..

[193] Srivastava V., Deblais L., Huang H.C., Miyazaki A., Kandasamy S., Langel S.N., Paim F.C., Chepngeno J., Kathayat D., Vlasova A.N. (2020). Reduced rotavirus vaccine efficacy in protein malnourished human-faecal-microbiota-transplanted gnotobiotic pig model is in part attributed to the gut microbiota. Benef. Microbes.

[194] WHO Malnutrition. https://www.who.int/news-room/fact-sheets/detail/malnutrition.

[195] Kandasamy S., Chattha K.S., Vlasova A.N., Saif L.J. (2014). Prenatal vitamin A deficiency impairs adaptive immune responses to pentavalent rotavirus vaccine (RotaTeq^®^) in a neonatal gnotobiotic pig model. Vaccine.

[196] Mora J.R., Iwata M., Eksteen B., Song S.Y., Junt T., Senman B., Otipoby K.L., Yokota A., Takeuchi H., Ricciardi-Castagnoli P. (2006). Generation of gut-homing IgA-secreting B cells by intestinal dendritic cells. Science.

[197] Perez-Schael I., Salinas B., Tomat M., Linhares A.C., Guerrero M.L., Ruiz-Palacios G.M. (2007). Efficacy of the human rotavirus vaccine RIX4414 in malnourished children. J. Infect. Dis..

[198] Linhares A.C., Carmo K.B., Oliveira K.K., Oliveira C.S., Freitas R.B., Bellesi N. (2002). Nutritional status in relation to the efficacy of the rhesus-human reassortant, tetravalent rotavirus vaccine (RRV-TV) in infants from Belem, para state, Brazil. Rev. Inst. Med. Trop. São Paulo.

[199] Michael H., Langel S.N., Miyazaki A., Paim F.C., Chepngeno J., Alhamo M.A., Fischer D.D., Srivastava V., Kathayat D., Deblais L. (2020). Malnutrition Decreases Antibody Secreting Cell Numbers Induced by an Oral Attenuated Human Rotavirus Vaccine in a Human Infant Fecal Microbiota Transplanted Gnotobiotic Pig Model. Front. Immunol..

[200] Miyazaki A., Kandasamy S., Michael H., Langel S.N., Paim F.C., Chepngeno J. (2018). Protein deficiency reduces efficacy of oral attenuated human rotavirus vaccine in a human infant fecal microbiota transplanted gnotobiotic pig model. Vaccine.

[201] Appaiahgari M.B., Glass R., Singh S., Taneja S., Rongsen-Chandola T., Bhandari N., Mishra S., Vrati S. (2014). Transplacental rotavirus IgG interferes with immune response to live oral rotavirus vaccine ORV-116E in Indian infants. Vaccine.

[202] Moon S.S., Tate J.E., Ray P., Dennehy P.H., Archary D., Coutsoudis A., Bland R., Newell M.L., Glass R.I., Parashar U. (2013). Differential profiles and inhibitory effect on rotavirus vaccines of nonantibody components in breast milk from mothers in developing and developed countries. Pediatr. Infect. Dis. J..

[203] Becker-Dreps S., Vilchez S., Velasquez D., Moon S.S., Hudgens M.G., Zambrana L.E., Jiang B. (2015). Rotavirus-specific IgG antibodies from mothers’ serum may inhibit infant immune responses to the pentavalent rotavirus vaccine. Pediatr. Infect. Dis. J..

[204] Rongsen-Chandola T., Strand T.A., Goyal N. (2014). Effect of withholding breastfeeding on the immune response to a live oral rotavirus vaccine in North Indian infants. Vaccine.

[205] Groome M.J., Moon S.S., Velasquez D., Jones S., Koen A., van Niekerk N., Jiang B., Parashar U.D., Madhi S.A. (2014). Effect of breastfeeding on immunogenicity of oral live-attenuated human rotavirus vaccine: A randomized trial in HIV-uninfected infants in Soweto, South Africa. Bull. World Health Organ.

[206] Ali A., Kazi A.M., Cortese M.M., Fleming J.A., Moon S., Parashar U.D., Jiang B., McNeal M.M., Steele D., Bhutta Z. (2015). Impact of withholding breastfeeding at the time of vaccination on the immunogenicity of oral rotavirus vaccine—A randomized trial. PLoS ONE.

[207] Mwila K., Chilengi R., Simuyandi M., Permar S.R., Becker-Dreps S. (2017). Contribution of Maternal Immunity to Decreased Rotavirus Vaccine Performance in Low- and Middle-Income Countries. Clin. Vaccine Immunol..

[208] Emperador D.M., Velasquez D.E., Estivariz C.F., Lopman B., Jiang B., Parashar U., Anand A., Zaman K. (2016). Interference of monovalent, bivalent, and trivalent oral poliovirus vaccines on monovalent rotavirus vaccine immunogenicity in rural Bangladesh. Clin. Infect. Dis..

[209] Steele A.D., De Vos B., Tumbo J., Reynders J., Scholtz F., Bos P., de Beer M.C., Van der Merwe C.F., Delem A. (2010). Co-administration study in South African infants of a live-attenuated oral human rotavirus vaccine (RIX4414) and poliovirus vaccines. Vaccine.

[210] Kaslow D.C. (2021). Force of infection: A determinant of vaccine efficacy?. NPJ Vaccines.

[211] Bennett A., Nagelkerke N., Heinsbroek E., Premkumar P.S., Wnęk M., Kang G., French N., Cunliffe N.A., Bar-Zeev N., Lopman B. (2017). Estimating the incidence of rotavirus infection in children from India and Malawi from serial anti-rotavirus IgA titres. PLoS ONE.

[212] Armah G., Lewis K.D., Cortese M.M., Parashar U.D., Ansah A., Gazley L., Victor J.C., McNeal M.M., Binka F., Steele A.D. (2016). A Randomized, Controlled Trial of the Impact of Alternative Dosing Schedules on the Immune Response to Human Rotavirus Vaccine in Rural Ghanaian Infants. J. Infect. Dis..

[213] Bines J.E., Thobari. J., Satria C.D., Handley A., Watts E., Cowley D., Nirwati H., Ackland J., Standish J., Justice F. (2018). Human Neonatal Rotavirus Vaccine (RV3-BB) to Target Rotavirus from Birth. N. Engl. J. Med..

[214] Mengelle C., Mansuy J.M., Prere M.F., Grouteau E., Claudet I., Kamar N., Huynh A., Plat G., Benard M., Marty N. (2013). Simultaneous detection of gastrointestinal pathogens with a multiplex Luminex-based molecular assay in stool samples from diarrhoeic patients. Clin. Microbiol. Infect..

[215] Eibach D., Krumkamp R., Hahn A., Sarpong N., Adu-Sarkodie Y., Leva A., Käsmaier J., Panning M., May J., Tannich E. (2016). Application of a multiplex PCR assay for the detection of gastrointestinal pathogens in a rural African setting. BMC Infect. Dis..

[216] Kotloff K.L., Nataro J.P., Blackwelder W.C., Nasrin D., Farag T.H., Panchalingam S., Wu Y., Sow S.O., Sur D., Breiman R.F. (2013). Burden and aetiology of diarrhoeal disease in infants and young children in developing countries (the Global Enteric Multicenter Study, GEMS): A prospective, case-control study. Lancet.

[217] Platts-Mills J.A., Babji S., Bodhidatta L., Gratz J., Haque R., Havt A., McCormick B.J., McGrath M., Olortegui M.P., Samie A. (2015). Pathogen-specific burdens of community diarrhoea in developing countries: A multisite birth cohort study (MAL-ED). Lancet Glob. Health.

[218] Mokomane M., Tate J.E., Steenhoff A.P., Esona M.D., Bowen M.D., Lechiile K., Pernica J.M., Kasvosve I., Parashar U.D., Goldfarb D.M. (2018). Evaluation of the Influence of Gastrointestinal Coinfections on Rotavirus Vaccine Effectiveness in Botswana. Pediatr. Infect. Dis. J..

[219] Praharaj I., Platts-Mills J.A., Taneja S., Antony K., Yuhas K., Flores J., Cho I., Bhandari N., Revathy R., Bavdekar A. (2019). Diarrheal Etiology and Impact of Coinfections on Rotavirus Vaccine Efficacy Estimates in a Clinical Trial of a Monovalent Human-Bovine (116E) Oral Rotavirus Vaccine, Rotavac, India. Clin. Infect. Dis..

[220] Wallace T.C., Guarner F., Madsen K., Cabana M.D., Gibson G., Hentges E., Sanders M.E. (2011). Human gut microbiota and its relationship to health and disease. Nutr. Rev..

[221] Sonnenburg J.L., Backhed F. (2016). Diet-microbiota interactions as moderators of human metabolism. Nature.

[222] Subramanian S., Huq S., Yatsunenko T., Haque R., Mahfuz M., Alam M.A., Benezra A., DeStefano J., Meier M.F., Muegge B.D. (2014). Persistent gut microbiota immaturity in malnourished Bangladeshi children. Nature.

[223] Iturriza-Gómara M., Cunliffe N.A. (2017). The Gut Microbiome as Possible Key to Understanding and Improving Rotavirus Vaccine Performance in High–Disease Burden Settings. J. Infect. Dis..

[224] Naylor C., Lu M., Haque R., Mondal D., Buonomo E., Nayak U., Mychaleckyj J.C., Kirkpatrick B., Colgate R., Carmolli M. (2015). Environmental Enteropathy, Oral Vaccine Failure and Growth Faltering in Infants in Bangladesh. EBioMedicine.

[225] Harris V.C., Haak B.W., Handley S.A., Jiang B., Velasquez D.E., Hykes B.L., Droit L., Berbers G.A.M., Kemper E.M., van Leeuwen E.M.M. (2018). Effect of Antibiotic-Mediated Microbiome Modulation on Rotavirus Vaccine Immunogenicity: A Human, Randomized-Control Proof-of-Concept Trial. Cell Host Microbe.

[226] Center for Health Protection (CHP) Health Topics on Rotavirus Infection by the Department of Health, the Government of the Hong Kong Special Administrative Region. 5 July 2019. https://www.chp.gov.hk/en/healthtopics/content/24/38.html.

[227] UNICEF (2009). Diarrhoea: Why Children Are Still Dying and What Can Be Done.

[228] Shumetie G., Gedefaw M., Kebede A., Derso T. (2018). Exclusive breastfeeding and rotavirus vaccination are associated with decreased diarrheal morbidity among under-five children in Bahir Dar, northwest Ethiopia. Public Health Rev..

[229] Krawczyk A., Lewis M.G., Venkatesh B.T., Nair S.N. (2016). Effect of Exclusive Breastfeeding on Rotavirus Infection among Children. Indian J. Pediatr..

[230] Das S., Sahoo G.C., Das P., Singh U.K., Jaiswal A.K., Singh P., Kumar R., Kumar R. (2016). Evaluating the Impact of Breastfeeding on Rotavirus Antigenemia and Disease Severity in Indian Children. PLoS ONE.

[231] Shen J., Zhang B., Zhu S., Chen J. (2018). No direct correlation between rotavirus diarrhea and breast feeding: A meta-analysis. Pediatrics Neonatol..

[232] Salim H., Karyana I.P., Sanjaya-Putra I.G., Budiarsa S., Soenarto Y. (2014). Risk factors of rotavirus diarrhea in hospitalized children in Sanglah Hospital, Denpasar: A prospective cohort study. BMC Gastroenterol..

[233] Yang Y., Pei J., Qin Z., Wei L. (2019). Efficacy of probiotics to prevent and/or alleviate childhood rotavirus infections. J. Funct. Foods.

[234] Fijan S. (2014). Microorganisms with claimed probiotic properties: An overview of recent literature. Int. J. Environ. Res. Public Health.

[235] Kawahara T., Makizaki Y., Oikawa Y., Tanaka Y., Maeda A., Shimakawa M., Komoto S., Moriguchi K., Ohno H., Taniguchi K. (2017). Oral administration of Bifidobacterium bifidum G9-1 alleviates rotavirus gastroenteritis through regulation of intestinal homeostasis by inducing mucosal protective factors. PLoS ONE.

[236] Vlasova A.N., Kandasamy S., Chattha K.S., Rajashekara G., Saif L.J. (2016). Comparison of probiotic lactobacilli and bifidobacteria effects, immune responses and rotavirus vaccines and infection in different host species. Vet. Immunol. Immunopathol..

[237] Lazarus R.P., John J., Shanmugasundaram E., Rajan A.K., Thiagarajan S., Giri S., Babji S., Sarkar, Kaliappan P.S., Venugopal S. (2018). The effect of probiotics and zinc supplementation on the immune response to oral rotavirus vaccine: A randomized, factorial design, placebo-controlled study among Indian infants. Vaccine.

[238] Zeuthen L.H., Fink L.N., Frøkiaer H. (2008). Toll-like receptor 2 and nucleotide-binding to ligomerization domain-2 play divergent roles in the recognition of gut-derived lactobacilli and bifidobacteria in dendritic cells. Immunology.

[239] Oksaharju A., Kankainen M., Kekkonen R.A., Lindstedt K.A., Kovanen P.T., Korpela R. (2011). Probiotic Lactobacillus rhamnosus downregulates FCER1 and HRH4 expression in human mast cells. World J. Gastroenterol..

[240] Ahmadi E., Alizadeh-Navaei R., Rezai M.S. (2015). Efficacy of probiotic use in acute rotavirus diarrhea in children: A systematic review and meta-analysis. Casp. J. Int. Med..

[241] Park M.S., Kwon B., Ku S., Ji G.E. (2017). The Efficacy of Bifidobacterium longum BORI and Lactobacillus acidophilus AD031 Probiotic Treatment in Infants with Rotavirus Infection. Nutrients.

[242] Chen S., Wanga Y., Lia P., Yina Y., Bijveldsa M.J., de Jongea H.R., Peppelenboscha M.P., Kainovb D.E., Pan Q. (2020). Drug screening identifies gemcitabine inhibiting rotavirus through alteration of pyrimidine nucleotide synthesis pathway. Antivir. Res..

[243] Dycke J.V., Arnoldi F., Papa G., Vandepoele J., Burrone O.R., Mastrangelo E., Tarantino D., Heylen E., Neyts J., Rocha-Pereira J. (2018). A Single Nucleoside Viral Polymerase Inhibitor against Norovirus, Rotavirus, and Sapovirus-Induced Diarrhea. J. Infect. Dis..

[244] Knorr J.I.E., Erro I.O., Andueza M.M.C., Blecua M.C.T., Martinez A.E., Iparraguirre A.I. (2008). Systematic review of the efficacy of racecadotril in the treatment of acute diarrhoea. An. Pediatr..

[245] La-Frazia S., Ciucci A., Arnoldi F., Coira M., Gianferretti P., Angelini M., Belardo G., Burrone O.R., Rossignol J.F., Santoro M.G. (2013). Thiazolides, a New Class of Antiviral Agents Effective against Rotavirus Infection, Target Viral Morphogenesis, Inhibiting Viroplasm Formation. J. Virol..

[246] Chen X., Liu L., Chen W., Qin F., Zhou F., Yang H. (2020). Ziyuglycoside II Inhibits Rotavirus Induced Diarrhea Possibly via TLR4/NF-κB Pathways. Biol. Pharm. Bull..

[247] Siddique I., Khan I. (2011). Mechanism of Regulation of Na-H Exchanger in Inflammatory Bowel Disease: Role of TLR-4 Signaling Mechanism. Dig. Dis. Sci..

[248] Chen S., Ding S., Yin Y., Xu L., Li P., Peppelenbosch M.P., Pan Q., Wang W. (2019). Suppression of pyrimidine biosynthesis by targeting DHODH enzyme robustly inhibits rotavirus replication. Antivir. Res..

[249] Huang H., Liao D., Zhou G., Zhu Z., Cui Y., Pu R. (2020). Antiviral activities of resveratrol against rotavirus in vitro and in vivo. Phytomedicine.

[250] Gordon M., Akobeng A. (2016). Racecadotril for acute diarrhoea in children: Systematic review and meta-analyses. Arch. Dis. Child..

[251] Mahapatro S., Mahilary N., Satapathy A.K., Das R.R. (2017). Nitazoxanide in Acute Rotavirus Diarrhea: A Randomized Control Trial from a Developing Country. J. Trop. Med..

[252] Eichwald C., De Lorenzo G., Schraner E.M., Papa G., Bollati M., Swuec P., de Rosa M., Milani M., Mastrangelo E., Ackermann M. (2018). Identification of a Small Molecule That Compromises the Structural Integrity of Viroplasms and Rotavirus Double-Layered Particles. J. Virol..

[253] Kim J.H., Kim K., Kim W. (2020). Genipin inhibits rotavirus-induced diarrhea by suppressing viral replication and regulating inflammatory responses. Sci. Rep..

[254] Center for Disease Control and Prevention (CDC) Rotavirus. Last Reviewed 23 April 2018. https://www.cdc.gov/rotavirus/index.html.

